# Polyglycerol‐Amine Covered Nanosheets Target Cell‐Free DNA to Attenuate Acute Kidney Injury

**DOI:** 10.1002/advs.202300604

**Published:** 2023-06-05

**Authors:** Kefei Wu, Xiaohui Lu, Yi Li, Yating Wang, Ming Liu, Hongyu Li, Huiyan Li, Qinghua Liu, Dan Shao, Wei Chen, Yi Zhou, Zhaoxu Tu, Haiping Mao

**Affiliations:** ^1^ Department of Nephrology， The First Affiliated Hospital， Sun Yat‐sen University NHC Key Laboratory of Clinical Nephrology Guangdong Provincial Key Laboratory of Nephrology Guangzhou Guangdong 510080 China; ^2^ Department of Otolaryngology the Sixth Affiliated Hospital of Sun Yat‐sen University Guangzhou Guangdong 510655 China; ^3^ School of Biomedical Sciences and Engineering South China University of Technology Guangzhou International Campus Guangzhou Guangdong 511442 China

**Keywords:** acute kidney injury, cell‐free DNA scavenging, functionalized nanosheets, neutrophil extracellular traps, polyglycerol‐amine

## Abstract

Increased levels of circulating cell‐free DNA (cfDNA) are associated with poor clinical outcomes in patients with acute kidney injury (AKI). Scavenging cfDNA by nanomaterials is regarded as a promising remedy for cfDNA‐associated diseases, but a nanomaterial‐based cfDNA scavenging strategy has not yet been reported for AKI treatment. Herein, polyglycerol‐amine (PGA)‐covered MoS_2_ nanosheets with suitable size are synthesized to bind negatively charged cfDNA in vitro, in vivo and ex vivo models. The nanosheets exhibit higher cfDNA binding capacity than polymer PGA and PGA‐based nanospheres owing to the flexibility and crimpability of their 2D backbone. Moreover, with low cytotoxicity and mild protein adsorption, the nanosheets effectively reduced serum cfDNA levels and predominantly accumulated in the kidneys to inhibit the formation of neutrophil extracellular traps and renal inflammation, thereby alleviating both lipopolysaccharide and ischemia‐reperfusion induced AKI in mice. Further, they decreased the serum cfDNA levels in samples from AKI patients. Thus, PGA‐covered MoS_2_ nanosheets can serve as a potent cfDNA scavenger for treating AKI and other cfDNA‐associated diseases. In addition, this work demonstrates the pivotal feature of a 2D sheet‐like structure in the development of the cfDNA scavenger, which can provide a new insight into the future design of nanoplatforms for modulating inflammation.

## Graphical abstract

Cell‐free DNA (cfDNA) released from damaged tubular epithelial cells, acts as a signaling molecule and causes kidney injury by neutrophil extracellular trap formation in acute kidney injury (AKI). Polyglycerol‐amine covered MoS_2_ nanosheets effectively attenuate AKI, at least, by directly scavenging of cfDNA.

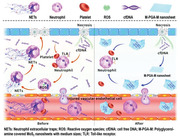

## 1 Introduction

Acute kidney injury (AKI) is characterized by an abrupt decline in renal function with high morbidity and mortality.^[^ ^1^, ^2^ ^]^ Further, when the damage is persistent, AKI can result in the development or progression of chronic kidney disease.^[^ ^3^, ^4^ ^]^ To date, there is a lack of effective treatment for AKI because of its complex and multifactorial etiology.

Cell‐free DNA (cfDNA) released by damaged or dead cells acts as a damage‐associated molecular pattern (DAMP).^[^ ^5^, ^6^ ^]^ These signaling molecules induce neutrophil extracellular traps (NETs),^[^ ^7^, ^8^ ^]^ which are implicated in many diseases.^[^ ^9^ ^]^ NETs are web‐like structures composed of decondensed chromatin histones and granular proteins, such as proteases, myeloperoxidase, and neutrophil elastase. The NET components can trigger a fatal circle inflammation contributing to organ injury and fatality.^[^ ^10^, ^11^ ^]^ Evolutionarily, the major function of platelets is to manage vascular integrity and regulate hemostasis. Recently, increasing evidences have indicated that interaction of platelet with neutrophils is indispensable in NET formation under pathological conditions.^[^ ^12^ ^]^ In animal models of ischemic AKI, cfDNA plays a crucial role in intrarenal inflammation, kidney damage,^[^ ^12^, ^13^, ^14^, ^15^ ^]^ and organ dysfunction^[^ ^16^ ^]^ through activating platelets and dysregulating NET formation, whereas inhibition of NET formation conferred renal protection.^[^ ^17^ ^]^ Further, serum cfDNA levels were markedly elevated and predicted poor prognosis in patients with AKI.^[^ ^18^, ^19^, ^20^ ^]^ NET formation was found in human renal allograft biopsies with acute tubular necrosis.^[^ ^16^ ^]^ Thus, increased cfDNA and NET formation not only lead to a consequence of AKI, but also amplify the damaging processes.^[^ ^21^ ^]^ Therefore, eliminating cfDNA and NETs could be a promising therapeutic approach for AKI treatment.^[^ ^12^ ^]^

Deoxyribonuclease (DNase) I has been shown to efficiently reduce the levels of cfDNA and NETs and treat cfDNA‐associated diseases,^[^ ^22^, ^23^ ^]^ but its clinical application is largely restricted by its side effects.^[^ ^24^ ^]^ Plasma exchange (PE) is an effective treatment for a variety of disease that removes and replaces patient's plasma components. Given that PE has a mass cut‐off of 3000 kDa,^[^ ^25^ ^]^ and cfDNA is fragmented in the range of 40–200 base pairs,^[^ ^26^ ^]^ assuming PE is competent for removing cfDNA. However, the roles of PE in clearance of cfDNA have not yet been reported that need to be further elucidated. With the rapid progress of biomaterial technology, cfDNA‐scavenging cationic nanomaterials have become a novel therapeutic strategy against cfDNA‐related diseases.^[^ ^27^, ^28^, ^29^, ^30^, ^31^ ^]^ However, challenges need to be addressed, including non‐specific protein adsorption, biodistribution and inflammation‐targeted properties, and toxicity.

Hyperbranched polyglycerol (hPG), synthesized via anionic ring‐opening multi‐branching polymerization, exhibits excellent biocompatibility, chemical stability, protein resistance, and tunable blood circulation.^[^ ^32^ ^]^ Because of negatively charged cfDNA and NETs,^[^ ^33^, ^34^, ^35^ ^]^ the hydroxyl groups on hPG were converted into positively charged amine groups, which can enhance its binding capacity.^[^ ^32^ ^]^ However, hPG molecules are quickly excreted by the kidneys owing to their small size (below 10 nm), which reduces their efficacy in targeting injured tissues. Fortunately, 2D nanosheets with an appropriate size (100–500 nm) have the potential to target inflamed kidneys via the enhanced permeability and retention (EPR) effect.^[^ ^36^, ^37^, ^38^ ^]^ Molybdenum disulfide (MoS_2_) is recognized as a promising 2D material because of its outstanding biocompatibility and moderate biodegradable rate in physiological conditions, potentially leading to clinical translation.^[^ ^39^ ^]^ Therefore, in our study, we conjugated hPG‐amine (PGA) onto MoS_2_ as a new strategy for cfDNA scavenging and modulating renal inflammation.

We synthesized PGA‐covered MoS_2_ nanosheets with different sizes and examined their therapeutic potential in AKI. The size that demonstrated robust cfDNA binding ability, mild protein adsorption, renal targeting, and low cytotoxicity was selected for subsequent investigation. Furthermore, the cfDNA scavenging features were also compared between the PGA‐covered nanosheets and nanospheres to clarify the role of the 2D nanostructure. Finally, we studied the efficacy and renal protective impact of this cfDNA scavenger in vitro, in vivo and ex vivo models.

## 2 Results and Discussion

### 2.1 Increased NET Markers in AKI Patients

**Figure 1:**
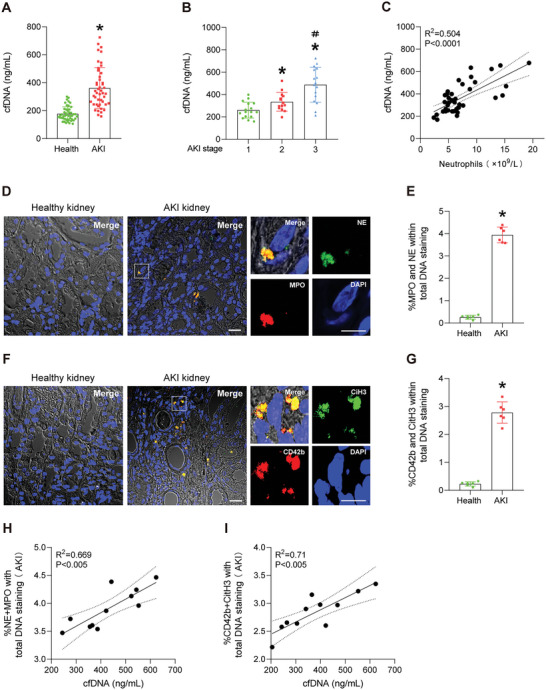
A) Serum levels of cfDNA in healthy volunteers (*n* = 47) and AKI patients (*n* = 46). B) Serum levels of cfDNA in patients with different stages of AKI. C) Correlation between serum cfDNA level and peripheral blood neutrophil count. D) Co‐immunostaining for myeloperoxidase (MPO, red) and neutrophil elastase (NE, green) in kidney biopsies from healthy and AKI patients. Scale bars: 20 µm. E) Percentage of MPO^+^NE^+^ cells of the total number of DAPI‐positive nuclei in (D). F) Co‐immunostaining for CD42b (red) with CitH3 (green) in kidney biopsies from healthy and AKI patients. Scale bars: 20 µm. G) Percentage of CD42b^+^CitH3^+^ cells of the total number of DAPI‐positive nuclei in (F). H) Positive correlation between the serum cfDNA level and the percentage of renal MPO^+^NE^+^ cells in all AKI patients. I) Positive correlation between serum cfDNA level and the percentage of CD42b^+^CitH3^+^ cells in all AKI patients. The data are expressed as the means ± SD and the categorical variables are presented as numbers (%). Statistical differences were examined using the unpaired Student's t‐test (A), one‐way ANOVA (B), and Mann‐Whitney U test (E, G) *p < 0.05, compared with healthy or stage 1; ＃*p* < 0.05, compared with stage 2.

A total of 46 patients with AKI and 47 healthy controls were included in this study. The process of patient selection and clinical characteristics are summarized in Figure S1 and Table S1 (Supporting Information). Patients with AKI had significantly higher values of serum creatinine (341.09 ± 195.48 *vs*. 69.77 ± 11.81 µmol L^−1^) (Table S1, Supporting Information) and cfDNA (362.51 ± 145.89 *vs*. 177.40 ± 50.21 ng mL^−1^) than the healthy controls (**Figure** **1**A). In line with previous findings that sepsis was the most common causes of AKI, and those patients had the highest levels of serum cfDNA (Table S1, Supporting Information**)**. Further, as AKI progressed, the serum cfDNA levels elevated markedly (Figure 1B). Correlation analysis showed a significant positive association between serum cfDNA level and absolute neutrophil count (Figure 1C). NET formation in the kidneys, as determined by immunofluorescent of myeloperoxidase and neutrophil elastase co‐staining was prominent in AKI patients, whereas myeloperoxidase and neutrophil elastase were not detected in healthy controls (Figure 1D,E). In addition, colocalization of citrullinated histone H3 (CitH3), which is a specific biomarker of NETs, and CD42b, which is an active form of platelets, was also evident in the kidneys of AKI patients, as compared with the healthy controls (Figure 1F,G). Moreover, the percentages of NETs (Figure 1H) and platelet‐neutrophil aggregates (Figure 1I) in the kidneys were both positively associated with the serum cfDNA levels in the AKI patients. Based on our results and those of previous studies in animal and in vitro models,^[^ ^13^, ^40^ ^]^ we speculated that increased circulating cfDNA might contribute to the pathogenesis of AKI through NET formation.

### 2.2 Synthesis and Characterization of PGA‐Covered MoS_2_ Nanosheets with Different Sizes

Previous studies in animal models demonstrated that scavenging cfDNA by cationic nanomaterials have a therapeutic effect in treating cfDNA‐associated diseases, including sepsis, rheumatoid arthritis and inflammatory bowel disease.^[^ ^27^, ^31^, ^41^ ^]^ However, the effect of nanomaterial‐based cfDNA scavenging in AKI has not been explored, and several challenges remain to be overcome before this can be effectively implemented. For example, most cationic nanomaterials show non‐specific protein adsorption that reduces the cfDNA‐scavenging capacity.^[^ ^42^ ^]^ Cationic polymers usually display unfavorable biodistribution and inflammation‐targeted properties that dampens the anti‐inflammatory performance. Finally, cationic polymers, including nanomaterials, exhibit high toxicity that hinders their biomedical application.

**Scheme 1:**
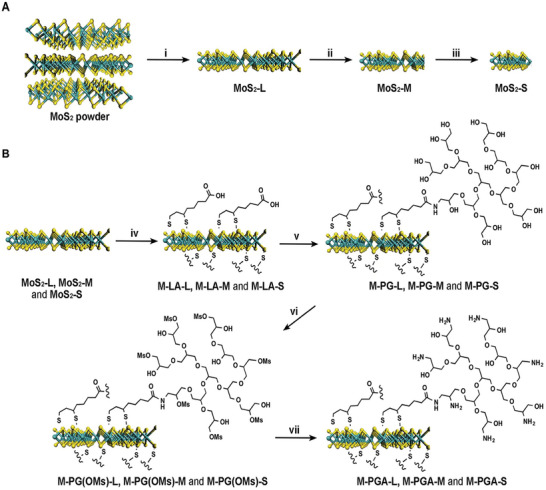
Synthesis routes of PGA‐covered MoS_2_ with different sizes. A) MoS_2_ monolayers with a large size (MoS_2_‐L) were prepared through intercalation with lithium ions, and were then broken down by sonication into medium (MoS_2_‐M) and small (MoS_2_‐S) sizes. B) PGA‐covered MoS_2_ with a large size (M‐PGA‐L), medium size (M‐PGA‐M) and small size (M‐PGA‐S) were synthesized through amidation, substitution and reduction reactions.

**Figure 2:**
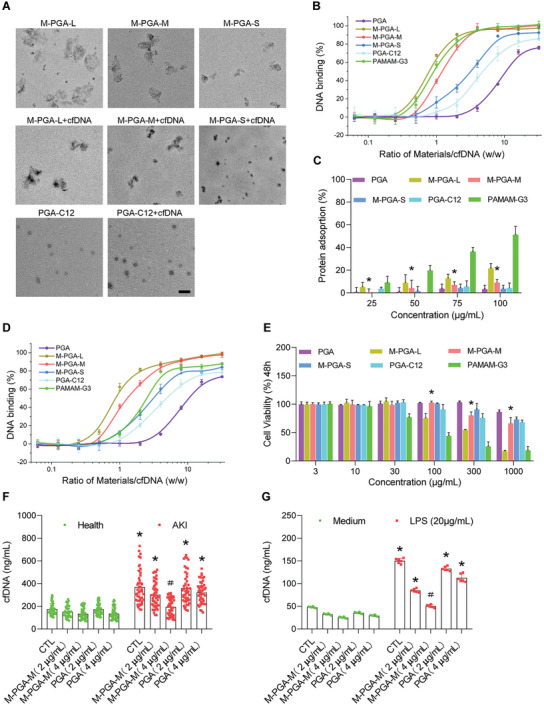
Characterization of cfDNA binding with PGA‐covered MoS_2_ nanosheets. A) Transmission electron microscopy images of PGA‐covered MoS_2_ nanosheets with different sizes. The nanosheets absorbed DNA extracted from fetal bovine thymus, and then corrugated and folded to form nanoparticles. Scale bars: 500 nm. B–D) Comparison of cfDNA binding efficiency (B), protein adsorption (C) and cfDNA binding efficiency (D) in FBS (10%) medium with PGA, M‐PGA‐L, M‐PGA‐M, M‐PGA‐S, PAMAM G3 and PGA‐C12. E) Viability of HK‐2 cells treated for 48 h with various concentrations of M‐PGA‐L, M‐PGA‐M, M‐PGA‐S, PGA, PAMAM G3 and PGA‐C12. The differences were assessed via Kruskal‐Wallis and Mann‐Whitney U tests (B, D), and one‐way ANOVA with Tukey's multiple comparison test (C, E), (*p < 0.05, compared with M‐PGA‐L or PAMAM‐G3). F,G) Serum cfDNA levels of healthy volunteers and AKI patients, as well as supernatant cfDNA of HK‐2 cells‐treated with LPS after incubation with either PGA or M‐PGA‐M for 30 min. The data are expressed as the means ± SD and the categorical variables are presented as percentages. The differences were assessed via one‐way ANOVA with Tukey's multiple comparison test (*p < 0.05, compared with a healthy or medium control (CTL); ＃*p* < 0.05, compared with AKI or LPS‐stimulated cells in the other treated groups).

In the pursuit of the optimal nanomaterial to scavenge cfDNA, we selected hyperbranched polyglycerol (hPG) and modified it with amino groups to synthesize positively charged hPG‐amine (PGA), which can adsorb negatively charged cfDNA and NETs via the electrostatic force.^[^ ^33^, ^34^, ^35^ ^]^ The protein‐resistant properties of hPG, and polyglycerol‐modified nanomaterials have been widely recognized due to their improved interaction with water molecules. As a result, proteins are unable to displace water molecules around the polymers and nanomaterials.^[^ ^43^ ^]^ In our study, hPG was synthesized via anionic ring‐opening multi‐branching polymerization (ROMBP) according to published methods.^[^ ^44^ ^]^ Subsequently, 50% of the hydroxyl groups on hPG were converted into amine groups to prepare PGA.^[^ ^32^ ^]^ MoS_2_ monolayers with a large size (MoS_2_‐L) were prepared through intercalation with lithium ions. MoS_2_‐L was then broken down with sonication using a tip transducer to form medium size (MoS_2_‐M) and small size (MoS_2_‐S) monolayers.^[^ ^32^ ^]^ To enhance cfDNA scavenging, PGA‐modified MoS_2_ with different sizes (M‐PGA‐L, M‐PGA‐M and M‐PGA‐S) were synthesized through a three‐step reaction (**Scheme** **1**).^[^ ^37^ ^]^ To determine whether the 2D sheet‐like structure plays a vital role in cfDNA scavenging, dodecanoic acid was conjugated onto PGA to prepare spherical PGA‐C12 nanoparticles for comparison (Figure S2, Supporting Information). The nuclear magnetic resonance (NMR) spectra (Figures S3 and S4, Supporting Information) and Fourier transform infrared (FTIR) spectrometry results (Figures S5 and S6, Supporting Information) confirmed the successful synthesis of PGA‐covered nanosheets and nanospheres.^[^ ^32^ ^]^ Transmission electron microscopy (TEM) images (**Figure** **2**A; Figure S7, Supporting Information) of the PGA‐modified nanosheets with different sizes revealed that they adsorbed cfDNA, and then corrugated and folded to form nanoparticles, which facilitated cfDNA scavenging. By contrast, this was not observed for the PGA‐C12 nanospheres. The size and zeta potential of M‐PGA‐L, M‐PGA‐M, M‐PGA‐S and PGA‐C12 were characterized, respectively (Figure S8, Supporting Information). In addition, the PGA content in M‐PGA‐L, M‐PGA‐M and M‐PGA‐S was calculated as 79.4%, 82.6% and 84.3%, respectively, from the UV–vis adsorption results (Figure S9, Supporting Information). To study the biodegradability of the nanomaterials, MoS_2_ nanosheets with and without the PGA coating were incubated in phosphate buffered saline (PBS) at 37 °C for 4 weeks. The UV absorption at a wavelength of 360 nm revealed that PGA‐covered MoS_2_ (in particular, those with the smallest size) degraded at a faster rate than bare MoS_2_ nanosheets (Figures S10 and S11, Supporting Information). Thus, the PGA‐coating improves the biosafety of the MoS_2_ nanosheets.

### 2.3 cfDNA Scavenging Efficiency and Cytotoxicity of M‐PGA‐M

We used calf thymus DNA to test the binding capacities of M‐PGA‐L, M‐PGA‐M, M‐PGA‐S and PGA‐C12 nanomaterials for cfDNA scavenging, as previous reported.^[^ ^29^ ^]^ We found that the nanosheets corrugated and bent after incubation with cfDNA, which was not observed for PGA‐C12 nanospheres (Figure 2A). Quantitative analysis revealed that the cfDNA binding capacity of PGA‐covered nanosheets was considerably higher than that of polymer PGA and PGA‐based nanospheres (Figure 2B), confirming our hypothesis a 2D geometry is advantageous for cfDNA scavenging by nanomaterials. Further, the cfDNA binding affinity elevated with increasing of the PGA‐covered nanosheet sizes, which was probably because of the larger sheet size and the higher surface zeta potential. In addition, PGA, M‐PGA‐S, M‐PGA‐M, M‐PGA‐L, and PGA‐C12 exhibited much lower adsorption of bovine serum albumin as compared with the commercial cationic polymer polyamindoamine generation 3 (PAMAM‐G3) (Figure 2C), which can be attributed to the protein‐resistance characteristics of hPG.^[^ ^45^ ^]^ Importantly, the presence of 10% fetal bovine serum markedly decreased the cfDNA binding efficacy of PAMAM‐G3, but not that of PGA‐covered nanoparticles (Figure 2D). Collectively, these data suggest the effectiveness of PGA‐covered MoS_2_ nanosheets in scavenging cfDNA with low non‐specific protein adsorption.

We next investigated the cytotoxicity of PGA‐covered nanosheets and nanospheres in vitro. PAMAM‐G3 at a concentration of 30 µg mL^−1^ caused a significant reduction in the viability of human renal proximal tubular cells (HK‐2 cells) at 48 and 72 h after exposure. By contrast, M‐PGA‐L, M‐PGA‐M, M‐PGA‐S and PGA‐C12 exhibited cytotoxicity at much higher doses of 300 and 1000 µg mL^−1^, respectively. Notably, M‐PGA‐M exhibited lower cytotoxicity than M‐PGA‐L or PAMAM‐G3 at doses of 100, 300 and 1000 µg mL^−1^ (Figure 2E; Figure S12, Supporting Information). These results revealed a size‐dependent cytotoxicity of PGA‐covered nanosheets, in which larger nanosheets had greater toxicity. Based on the protein‐adsorption properties of PGA‐covered nanosheets and nanospheres in cfDNA binding and their cytotoxicity, M‐PGA‐M was selected for the following investigation. To ensure the safety of M‐PGA‐M, an in vitro hemocompatibility assay was performed with fresh red blood cells from adult donors. The hemolysis rates of M‐PGA‐M and PGA were lower than 5% at concentrations ranging from 1 to 100 µg mL^−1^ after 24 h exposure, which is defined as non‐hemolytic according to the ASTM F756‐2008 standard^[^ ^46^ ^]^ (Figure S13A, Supporting Information). Exposure to M‐PGA‐M or PGA did not affect the morphology of the red blood cells (Figure S13B, Supporting Information). Also, there was no apparent influence on the morphology of human platelets and neutrophils when compared before and after treatment (Figure S13C, Supporting Information), suggesting good blood compatibility of M‐PGA‐M. In addition, HK‐2 cells phalloidin staining showed that, compared with the vehicle control, neither M‐PGA‐M nor PGA treatments altered the actin cytoskeleton morphology (Figure S13D, Supporting Information), indicating no apparent cytotoxicity. Taken together, these results indicate that PGA‐covered MoS_2_ nanosheets with a medium size are highly biocompatible nanosheets appropriate for in vivo administration.

Up to date, cfDNA scavenging by cationic biomaterials has only been conducted in animal models or in vitro as reported in the literature. We thus explored whether M‐PGA‐M could scavenge cfDNA derived from samples of AKI patients or the supernatant cfDNA from HK‐2 cells treated with LPS. As shown in Figure 2F, a higher dose of M‐PGA‐M (4 µg mL^−1^) was more effective than a lower dose (2 µg mL^−1^) in reducing the elevated levels of serum cfDNA from AKI patients, whereas PGA had no such effect. In HK‐2 cells, LPS exposure increased supernatant cfDNA levels. Consistently, M‐PGA‐M, but not PGA, significantly and dose‐dependently scavenged supernatant cfDNA (Figure 2G). Collectively, our results further verified the importance of the 2D structure of M‐PGA‐M in cfDNA scavenging. These findings first demonstrated that M‐PGA‐M is effectively able to reduce serum samples cfDNA levels from AKI patients, suggesting the potential for this material to be applied in a clinical setting. Based on above results, 4 µg mL^−1^ of M‐PGA‐M was selected as the optimal concentration for subsequent experiments.

### 2.4 M‐PGA‐M Suppressed Human Platelet Activation and NET Formation In Vitro

**Figure 3:**
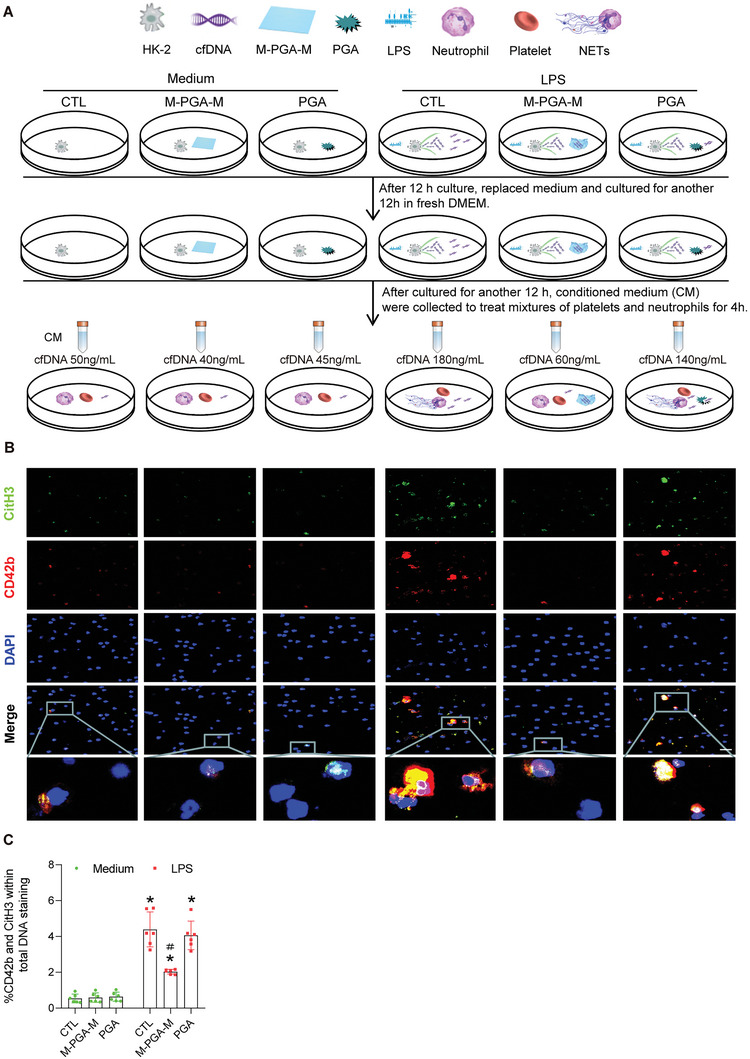
Effect of M‐PGA‐M on platelet activation and NET formation in vitro. A) Schematic diagram of the experimental design. Briefly, HK‐2 cells were concurrently treated with LPS or media containing the indicated agents (M‐PGA‐M and PGA). Twelve hours later, the medium was replaced, and the cells were cultured for an additional 12 h. Then, the conditioned medium with different cfDNA concentrations corresponding to the different treatment groups was collected to treat mixtures of platelets and neutrophils from healthy volunteers for 4 h. B) Representative confocal images of the cells treated in (A). Labels: nuclei (DAPI, blue), active platelets (CD42b, red) and neutrophils (CitH3, green). Scale bars: 20 µm. C) Percentage of CD42b^+^CitH3^+^ cells over DAPI. The data are expressed as percentages (*n* = 3). The differences were assessed via Kruskal–Wallis and Mann–Whitney U tests (*p < 0.05, compared with medium control (CTL); ＃*p* < 0.05, compared with LPS alone or LPS + PGA).

Given that cfDNA is a potent activator of platelets and because activated platelets interact with neutrophils, resulting in NET formation,^[^ ^13^, ^40^ ^]^ we hypothesized that M‐PGA‐M might inhibit human platelet activation and NET formation via scavenging cfDNA. To test this end, HK‐2 cells were stimulated with LPS in the presence or absence of M‐PGA‐M for 12 h. The medium was then replaced with fresh one and the cells were cultured for an additional 12 h. The conditioned medium was then collected and used to treat mixtures of platelets and neutrophils taken from healthy volunteers for 4 h. Consistent with the results shown in Figure 2G, stimulation of HK‐2 cells with LPS for 30 min caused a more than threefold increase in the levels of supernatant cfDNA compared with control cells. The addition of M‐PGA‐M, but not PGA, greatly decreased LPS‐induced cfDNA release (**Figure** **3**A). As expected, platelet–neutrophil aggregates, as evident by the colocalization of CD42b and CitH3 staining, were prominent after exposure to the conditioned medium from LPS‐treated HK‐2 cells (Figure 3B). By contrast, there were far fewer aggregates after exposure to the conditioned medium from cells treated with a combination of LPS and M‐PGA‐M. Notably, PGA appeared to decrease the number of platelet‐neutrophil aggregates but did not reach statistically significant (Figure 3B,C). NET formation, as demonstrated by Sytox Green staining, was strongly suppressed when the mixture was treated with conditioned medium from LPS‐stimulated cells in the presence of M‐PGA‐M, whereas PGA had no effect on NET formation (Figure S14A and B, Supporting Information). Similarly, the addition of M‐PGA‐M, but not PGA, significantly reduced the LPS‐triggered production of reactive oxygen species (ROS) (Figure S15A and B, Supporting Information) and malonaldehyde (Figure S15C, Supporting Information), and enhanced the activities of superoxide dismutase (Figure S15D, Supporting Information) and glutathione (Figure S15E, Supporting Information). Thus, our data revealed that M‐PGA‐M with a 2D nanostructure efficiently blocked platelet‐neutrophil aggregation, NET formation, and oxidative stress.

### 2.5 Biodistribution and Kidney‐Targeting Efficacy of M‐PGA‐M

**Figure 4:**
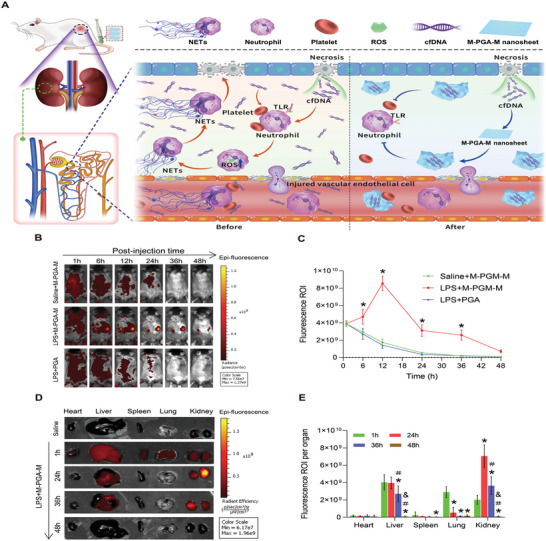
Biodistribution of M‐PGA‐M in vivo. A) Schematic illustration of the protection mechanism of M‐PGA‐M against LPS‐induced AKI in mice. M‐PGA‐M exhibited strong accumulation and retention in the injured kidneys, where it scavenged cfDNA, inhibited platelet activation and NET formation, thus attenuating LPS‐induced AKI in mice. B) Ex vivo fluorescence imaging of Cy5‐labeled M‐PGA‐M or PGA in saline‐ or LPS‐treated mice. C) Region of interest (ROI) analysis of the mice after different treatments (*p < 0.05, compared with saline + M‐PGA‐M or LPS+PGA). D) Ex vivo fluorescence imaging of Cy5‐labeled M‐PGA‐M in the major organs of saline‐ or LPS‐treated mice. E) Quantitative assessment of the distribution of Cy5‐labeled M‐PGA‐M in the major organs. The data are expressed as the means ± SD (*n* = 6). The differences were assessed via one‐way ANOVA with Tukey's multiple comparison test (*p < 0.05, compared with 1 hour; ＃*p* < 0.05, compared with 24 h; & *p* < 0.05, compared with 36 h).

M‐PGA‐M is expected to accumulate via the EPR effect, thereby alleviating LPS‐induced kidney damage by scavenging cfDNA and disturbing NET formation (**Figure** **4**A). To assess the distribution and accumulation of cationic M‐PGA‐M in the kidneys of LPS‐induced AKI mice, the nanosheets were labeled with hydrophilic Cy5 (Figure 4B). The fluorescent signals in the kidneys seemed to rise slightly at 6 h, peaked at 12 h, and lasted for at least 36 h post‐injection. The peak fluorescence intensity was 2.3‐fold higher than that in sham mice or AKI mice treated with PGA (Figure 4C). In contrast with the long retention of M‐PGA‐M in the kidneys of AKI mice, fluorescence was eliminated within 12 to 24 h in the other two groups (Figure 4B,C). Further, in AKI mice treated with M‐PGA‐M, ex vivo imaging of the excised major organs showed fluorescence signals in all organs and substantial accumulation in the kidneys at 1 h post‐injection, and then liver within 36 h, at which point signals were no longer discernible from the other organs (Figure 4D,E). Of note, the fluorescent signals appeared to widely distribute in renal glomerulus, tubular epithelial cells, and interstitium (Figure S16, Supporting Information). This might be explained that LPS can cause various compartments of the renal tissue damage, suggesting M‐PGA‐M preferably accumulated and distributed across the injured kidney.

Given the complexity of the renal structure and the heterogeneity of pathological features, it is of great significance to develop kidney‐targeted nanoparticles to achieve desired outcomes for AKI. The charge and size affect the accumulation and retention of nanoparticles in the kidneys. Nanoparticles with sizes >100 nm are generally too large to filter through the glomerular filtration barrier; however, these particles have been reported to enter into the kidneys via secretion from the peritubular capillaries to the proximal tubule.^[^ ^47^ ^]^ In our study, M‐PGA‐M were positively charged with a size of 249 ± 15 nm. The biodistribution results confirmed that, in the mouse model of LPS‐induced AKI, there was greater and longer accumulation of M‐PGA‐M than PGA in the injured kidneys. Thus, M‐PGA‐M with 2D nanosheets structure and an appropriate size accumulated in injured kidneys owing to the EPR effect,^[^ ^36^, ^37^, ^38^ ^]^ which is favorable for AKI treatment.

### 2.6 M‐PGA‐M Attenuates both LPS and Ischemia‐Reperfusion Induced AKI in Mice

**Figure 5:**
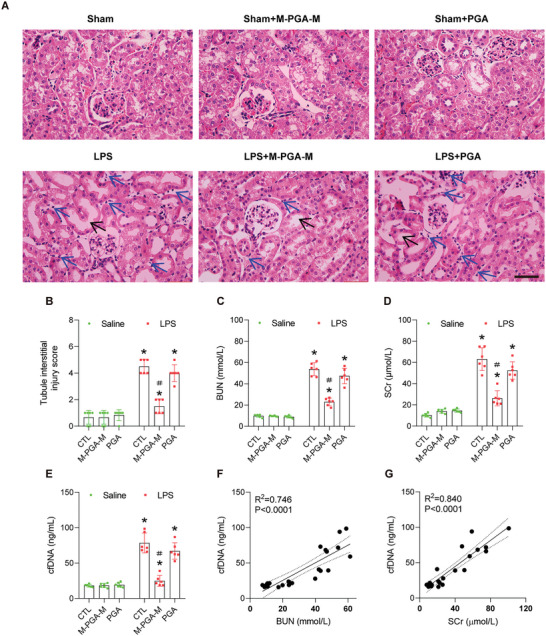
Amelioration of LPS‐induced AKI by M‐PGA‐M in vivo. A) Representative images of renal H&E staining. Scale bars: 50 µm. The black arrows indicate dilated tubules, the blue arrows indicate the infiltration of inflammatory cells. B) Quantification of tubulointerstitial damage. C–E) Changes in blood urea nitrogen (BUN) (C), serum creatinine (SCr) (D) and cfDNA (E) levels in LPS‐treated mice with or without M‐PGA‐M or PGA at 24 h. F) Correlation between the serum cfDNA and BUN levels. G) Correlation between the serum cfDNA and SCr levels. The data are expressed as the means ± SD (*n* = 6). The differences were assessed via one‐way ANOVA with Tukey's multiple comparison test (*p < 0.05, compared with saline; ＃*p* < 0.05, compared with LPS alone or LPS + PGA).

We sought to determine the potential therapeutic effect of M‐PGA‐M in LPS‐induced AKI in mice. Consistent with previous report, kidney sections of the LPS‐treated mice showed typical AKI histopathological changes, including tubular vacuolization, loss of brush border, tubular dilation and inflammatory cell infiltration compared with sham mice. Treatment with M‐PGA‐M, but not PGA, ameliorated these pathological changes (**Figure** **5**A and B**;** Figure S17, Supporting Information) and improved kidney function, as measured by blood urea nitrogen and serum creatinine (Figure 5C,D). To explore the effect of M‐PGA‐M on cfDNA scavenging, we measured the serum levels of cfDNA, which significantly increased in LPS‐induced AKI mice compared with vehicle and sham mice. Notably, M‐PGA‐M treatment reduced the cfDNA levels to normal levels, whereas PGA had no such effect (Figure 5E). Positive correlation was observed between the serum cfDNA level and renal function (Figure 5F,G). These data agreed with the in vitro cfDNA binding results, confirmed, and expanded the cfDNA‐scavenging ability of M‐PGA‐M in vivo. To substantiate our findings and rule out the plausible alternative that the protective effect of M‐PGA‐M might scavenging other deleterious non‐DNA molecules, pathogen‐derived unmethylated CpG dinucleotides, as a cfDNA surrogate^[^ ^49^, ^50^, ^51^, ^52^, ^53^, ^54^, ^55^ ^]^ was injected to LPS‐induced AKI mice via tail vein. As shown in Figure S18A (Supporting Information), compared with LPS challenge alone, in combination with CpG (LPS‐CpG) led to increased mortality rate (29% *vs*. 86%, *p* = 0.0224), indicating that cfDNA per se could aggravated damages of LPS‐induced AKI. In contrast, treatment with M‐PGA‐M significantly reduced LPS‐CpG‐induced mortality rate of mice from 86% to 29%, with the corresponding improvement in elevated serum cfDNA levels (Figure S18B, Supporting Information), renal pathological deterioration (Figure S18C and D, Supporting Information) and dysfunction (Figure S18E and F, Supporting Information). Collectively, our data revealed that M‐PGA‐M was able to rescue cfDNA‐mediated AKI in mice.

**Figure 6:**
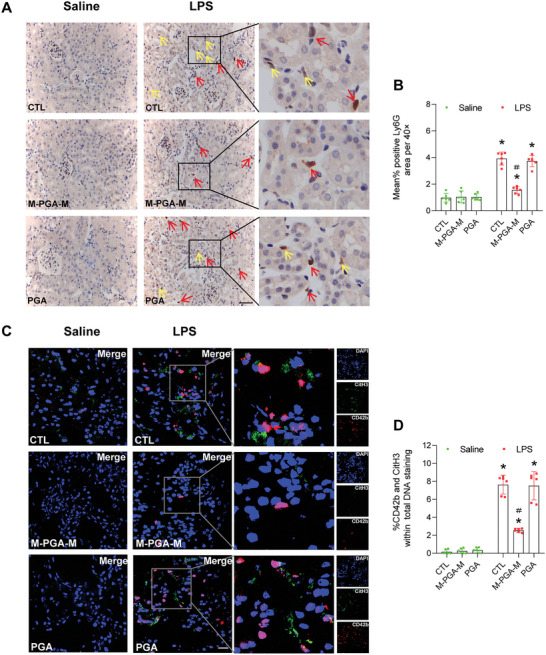
Inhibition of platelet activation and NET formation by M‐PGA‐M in vivo. A) Representative immunohistochemistry staining for Ly6G in the kidneys from different groups. Note the positive staining for Ly6G in the renal tubule (yellow arrows) and interstitium (red arrows). Scale bars: 200 µm. B) Quantification of Ly6G‐positive cells in the kidneys. C) Representative immunofluorescence staining for CD42b (red) and CitH3 (green) in the kidneys from different groups. DAPI (blue) indicates the nuclei. Scale bars: 20 µm. D) Percentage of CD42b^+^CitH3^+^ cells of the total number of DAPI positive nuclei in (C). The data are expressed as the means ± SD. The differences were assessed via Kruskal–Wallis and Mann–Whitney U tests (*p < 0.05, compared with saline control (CTL); ＃*p* < 0.05, compared with LPS‐treated alone or LPS + PGA).

LPS can trigger a system inflammatory response, we next investigated the role of M‐PGA‐M in the renal inflammation as well as the function of the liver and heart. As shown in **Figure** **6**A and B, upon LPS administration, neutrophil infiltration, as determined by lymphocyte antigen 6G (Ly6G) staining, significantly increased in the renal interstitium compared with the saline control group, and was markedly diminished under M‐PGA‐M treatment. By contrast, PGA failed to show a protective effect. Immunofluorescence staining showed that, compared with PGA, M‐PGA‐M treatment markedly inhibited platelet activation and NET formation (Figure 6C,D). Because LPS‐mediated oxidative stress is crucial in modulating renal inflammation, we expected that suppression of oxidation products would contribute to the therapeutic effect of M‐PGA‐M. Compared with LPS treatment alone, the administration of M‐PGA‐M greatly reduced the production of ROS, lowered malonaldehyde levels (Figure S19A–C, Supporting Information), and increased the levels of glutathione and superoxide dismutase (Figure S19D and E, Supporting Information) in the kidney following LPS injection. Moreover, serum levels of aspartate transaminase, alanine transaminase, creatine kinase, and creatine kinase‐MB were all reduced by M‐PGA‐M (Figure S20A–D, Supporting Information). Although we only examined serum biomarkers without pathological alterations, the results indicated ameliorative hepatic and cardiac injury by administration of M‐PGA‐M. LPS‐induced AKI is frequently accompanied by damage to renal endothelial cells and increased capillary permeability, as well as an uncontrolled inflammatory response.^[^ ^55^ ^]^ Therefore, we speculated that M‐PGA‐M might adsorb excessive cfDNA during circulation upon intravenous administration, gain access through peritubular capillaries to the tubule, gradually accumulate in renal inflammatory sites and ameliorate renal injury by scavenging cfDNA. In addition, negatively charged cfDNA and NETs in the kidneys induced by LPS may promote the accumulation of M‐PGA‐M in the renal tubulointerstitium. To confirm our findings, we induced AKI by ischemia‐reperfusion (IR) to further validate the renal protection of M‐PGA‐M. Consistently, treatment with M‐PGA‐M significantly mitigated the IR‐induced increase in BUN and Scr, histological scores and the cfDNA as compared with the untreated IR model mice. By contrast, either PGA or PGA‐C12 failed to protect kidney against IR injury and increased cfDNA levels (Figure S21, Supporting Information), suggesting that M‐PGA‐M is effective to inhibit AKI by cfDNA scavenging in vivo.

cfDNA can form a positive feedback loop with inflammation. This systemic response aggravates the initial organ damage, which, in turn, triggers a further vicious cycle of cfDNA, inflammation, and organ injury. Hence, simultaneously scavenging local and circulating cfDNA enables nanomaterials to achieve optimal efficacy. As we have shown above, polyglycerol‐amine (PGA)‐covered MoS_2_ nanosheets effectively reduced serum cfDNA levels in samples from AKI patients (Figure 2F ) and LPS‐induced AKI mice (Figure 5 ). Meanwhile, it actively targeted the inflamed organ through EPR effect (Figure 4 ), and thereby improving kidney, liver, and heart function (Figure S20, Supporting Information). Hence, our findings suggest the protective role of M‐PGA‐M against AKI through local and circulating cfDNA scavenging.

In summary, attributed to the appropriate material properties, shape, charge and size, M‐PGA‐M demonstrated strong multi‐organ protection, highlighting the therapeutic potential of this nanomedicine strategy for treating AKI. Nevertheless, we acknowledge some limitations of our study, including the absence of in vivo validation of M‐PGA‐M degradation and renal clearance. Further studies are required to comprehensively address the roles and mechanisms of M‐PGA‐M in treatment of AKI.

## 3 Conclusion

We fabricated PGA‐covered MoS_2_ nanosheets to scavenge cfDNA, which is a novel therapeutic target to treat AKI. The functionalized nanosheets with suitable size had mild cytotoxicity and low protein adsorption. Furthermore, their cfDNA binding was considerably higher than that of polymer PGA and the PGA‐based nanospheres because of the wrapping and folding properties of the 2D nanomaterial. The PGA‐covered MoS_2_ nanosheets were effective in reducing serum cfDNA levels in samples from AKI patients and mice. In addition, they had outstanding in vivo biocompatibility and renal retention in LPS‐induced AKI in mice, resulting in suppressed inflammatory response, attenuated renal damage and improved renal function compared with their PGA counterparts. Therefore, our findings demonstrate the significance of a 2D sheet‐like structure and the planar size in the design and fabrication of nanoplatforms for cfDNA scavenging and inflammation modulation, and provide a new strategy for the development of future scavenging approaches for the treatment of AKI.

## 4 Experimental Section

### Synthesis of hPG(NH_2_)_5%_

Hyperbranched polyglycerol (hPG) (*M*_n_ ≈ 5000 g mol^−1^) was synthesized via anionic ring‐opening multi‐branching polymerization according to previous publications.^[^ ^57^ ^]^ First, hPG (1 g, 0.2 mmol, 13.5 mmol hydroxyl groups) was dissolved in dimethylformamide (DMF, 10 mL) and stirred at room temperature (RT) for 1 h, prior to adding methanesulfonyl chloride (MsCl, 0.05 mL, 0.67 mmol, Fisher Scientific, USA) and triethylamine (TEA, 0.28 mL, 2 mmol, Fisher Scientific, USA). The reaction mixture was stirred in nitrogen at RT for 24 h, and then evaporated. The product was dialyzed in methanol (Fisher Scientific, USA) for 24 h to obtain purified hPG(OMs)_5%_. Next, hPG(OMs)_5%_ (0.8 g, 0.16 mmol) was dissolved in DMF (20 mL) and sodium azide (0.18 g, 2.7 mmol, Fisher Scientific, USA) was added to the mixture. The reaction was stirred at 80 °C for 24 h, and then hPG(N_3_)_5%_ as the product was purified by dialysis in methanol for 24 h. Finally, hPG(N_3_)_5%_ (0.5 g, 0.1 mmol) was dissolved in a mixture of tetrahydrofuran (THF, Fisher Scientific, USA) and Milli‐Q‐purified water (THF:H_2_O = 2:1), and triphenylphosphine (PPh3, 0.43 g, 1.6 mmol, Fisher Scientific, USA) was added to the solution. The reaction was conducted at 45 °C for 48 h to reduce azido groups to amino groups before the solvent was evaporated. The product was dialyzed against methanol for 24 h to obtain hPG(NH_2_)_5%_.

### Synthesis of MoS_2_ Monolayers with Different Sizes

MoS_2_ monolayers were prepared using lithium anions as intercalation agents according to a previous report.^[^ ^58^ ^]^ MoS_2_ powder (200 mg, Fisher Scientific, USA) was introduced to a dry Schlenk flask in an argon atmosphere, followed by the addition of n‐butyllithium (Fisher Scientific, USA) in n‐hexane (25 mL). The reaction was condensed and refluxed at 60 °C for 48 h. After centrifugation at 2000 rpm for 5 min, the supernatants were discarded, and the precipitate was collected. The products were precipitated by centrifugation, washed with n‐hexane, and further dispersed in Milli‐Q water to remove the unexfoliated sheets. After re‐dispersal, the products were dialyzed in pure water (molecular‐weight cut‐off (MWCO) = 12–14 KDa) for 3 days to obtain pure MoS_2_ monolayer sheets with a large size (MoS_2_‐L). In the next step, MoS_2_ sheets were dispersed in water (1 mg mL^−1^) and subjected to tip sonication in argon for 10 and 20 min to produce MoS_2_ sheets with medium (MoS_2_‐M) and small sizes (MoS_2_‐S), respectively.

### Synthesis of PGA‐Covered MoS_2_ with Different Sizes

To synthesize lipoic acid (LA)‐modified MoS_2_ (MoS_2_‐LA‐L), 1 mg mL^−1^ MoS_2_‐L in aqueous solution and 10 mg mL^−1^ LA (Fisher Scientific, USA) in methanol were prepared. Then, 0.5 mL LA solution was added to 10 mL MoS_2_‐L solution and stirred at RT for 24 h. MoS_2_‐LA‐L (100 mg) was collected and dissolved in 2‐(N‐morpholino) ethanesulfonic acid (MES, 10 mm, Fisher Scientific, USA) with *N*‐(3‐dimethylaminopropyl)‐Nʹ‐ethylcarb‐odiimide hydrochloride (EDC.HCl, 5 mm, Fisher Scientific, USA). hPG(NH_2_)_5%_ (1000 mg) was then added. The reaction solution was stirred at 37 °C for 48 h and then purified by dialysis (MWCO = 12–14 KDa) for 2 days to obtain hPG‐covered MoS_2_‐L (M‐hPG‐L). Approximately 50% of the hydroxyl groups on M‐hPG‐L were converted to amino groups to produce hPG‐amine (PGA)‐covered MoS_2_‐L (M‐PGA‐L) according to the protocol described above for hPG(NH_2_)_5%_ synthesis. PGA‐covered MoS_2_‐M (M‐PGA‐M) and PGA‐covered MoS_2_‐S (M‐PGA‐S) were prepared with MoS_2_‐M and MoS_2_‐S using the same protocol.

### Synthesis of PGA‐C12

50 mg (0.25 mmol) dodecanoic acid was dissolved in 100 mL DMF with 95.8 mg (0.5 mmol) EDC‐HCl and 57.5 mg (0.5 mmol) NHS. After that, 250 mg (0.05 mmol) PGA was added to the solution and allowed to react at RT for 24 h. The solution was dialysis in Milli‐Q (MWCO = 500) to remove unreacted agents and DMF, and was lyophilized to obtain PGA‐C12. To prepare PGA‐C12 nanoparticles, 50 mg of PGA‐C12 was dissolved in 5 mL DMSO and added dropwise to 45 mL water. DMSO in solution was then removed by dialysis in Milli‐Q water (MWCO = 500) for 24 h.

### Protein‐Adsorption Measurement

The protein‐adsorption test was performed according to a previous report.^[^ ^58^ ^]^ PGA, M‐PGA‐S, M‐PGA‐M, M‐PGA‐L, PGA‐C12 and PAMAM‐G3 solutions (1 mL, 100 µg mL^−1^) were mixed with bovine serum albumin (BSA, 1 mL, 100 µg mL^−1^). After stirring for 30 min at 37 °C, the mixture was centrifugated at 11000 rpm. The supernatant was carefully collected and the protein concentration was determined using the bicinchoninic acid protein assay. The protein adsorption capacity (PA) of PGA, M‐PGA‐S, M‐PGA‐M, M‐PGA‐L, PGA‐C12 and PAMAM‐G3 was calculated using the following equation: PA = (*C*_1_ − *C*_2_) × *V*/*m*.^[^ ^58^ ^]^ In this equation, *C*_1_ and *C*_2_ were the initial BSA concentration and the BSA concentration in the supernatant after centrifugation, respectively, *V* = 2 mL and *m* = 100 mg.

### cfDNA‐Binding Assay

Pico‐green fluorescent dye (Quant‐iT™ PicoGreen™ dsDNA Assay Kits, Fisher Scientific, USA) was used to determine the cfDNA binding efficiency of the nanosheets. Briefly, calf thymus DNA (1 µg mL^−1^, Sigma–Aldrich, USA) was mixed with Pico‐green dye (2 µg mL^−1^) and incubated in TE buffer at 37 °C for 30 min. Then, PGA, M‐PGA‐S, M‐PGA‐M, M‐PGA‐L, PGA‐C12 and PAMAM‐G3 with concentrations ranging from 0.0625 to 32 µg mL^−1^ were added, and the mixture was incubated at 37 °C for 30 min. The fluorescence intensities were analyzed with 480‐nm excitation and 520‐nm emission wavelengths.

### Biodegradation Test

The UV absorption of materials at 360 nm was used for the biodegradation tests. Briefly, MoS_2_‐L, MoS_2_‐M, MoS_2_‐S, M‐PGA‐L, M‐PGA‐M and M‐PGA‐S were dispersed in 1 mg mL^−1^ PBS (pH 7.4) and incubated at 37 °C for 4 weeks. The UV–vis absorption spectra were recorded on a U‐3310 spectrophotometer (Hitachi, Japan) after each week of incubation.

## Conflict of Interest

The authors declare no conflict of interest.

## Author Contributions

K.F.W., X.H.L., and Y.L. contributed equally to this work. M.L., D.S. and Z.X.T. synthesized and characterized the functional nanomaterials. K.F.W., X.H.L., and Y.L. performed all in vitro and animal experiments. Y.T.W., and H.Y.L. collected and analyzed clinical data. H.Y.L., and W.C. conducted kidney histologic examination. H.P.M., Z.X.T., Y.Z., and K.F.W. designed the study and wrote the manuscript.

## Supporting information

Supporting Information

## Data Availability

The data that support the findings of this study are available in the supplementary material of this article.
