# Supplementary material for: Polyglycerol‐Amine Covered Nanosheets Target Cell‐Free DNA to Attenuate Acute Kidney Injury
Source: Adv Sci (Weinh). 2023 Jun 5;10(23):2300604. doi: 10.1002/advs.202300604 (PMC10427348; doi:10.1002/advs.202300604)
Supplement: Supplementary file 1 — Supporting Information [file ADVS-10-2300604-s001.pdf]

ADVANCED SCIENCE
Open Access

Supporting Information

for *Adv. Sci.*, DOI 10.1002/advs.202300604

Polyglycerol-Amine Covered Nanosheets Target Cell-Free DNA to Attenuate Acute Kidney Injury

Kefei Wu, Xiaohui Lu, Yi Li, Yating Wang, Ming Liu, Hongyu Li, Huiyan Li, Qinghua Liu, Dan Shao, Wei Chen, Yi Zhou, Zhaoxu Tu* and Haiping Mao**

Supporting Information

Polyglycerol-amine Covered Nanosheets Target Cell-free DNA to Attenuate Acute Kidney Injury

Kefei Wu[1], Xiaohui Lu[1], Yi Li[1], Yating Wang[1], Ming Liu[2], Hongyu Li[1], Huiyan Li[1], Qinghua Liu[1], Dan Shao[3], Wei Chen[1], Yi Zhou[1], Zhaoxu Tu[2]*, and Haiping Mao[1]**

[1]Department of Nephrology, The First Affiliated Hospital, Sun Yat-sen University, NHC Key Laboratory of Clinical Nephrology, Guangdong Provincial Key Laboratory of Nephrology, Guangzhou, Guangdong, 510080, China

[2]Department of Otolaryngology, the Sixth Affiliated Hospital of Sun Yat-sen University, Guangzhou, Guangdong, 510655, China

[3]School of Biomedical Sciences and Engineering, South China University of Technology, Guangzhou International Campus, Guangzhou, Guangdong 511442, China

E-mail: maohp@mail.sysu.edu.cn; tuzhx@mail.sysu.edu.cn; zhouyi39@mail.sysu.edu.cn

Table of contents

1. Experimental details 4

2. Figure S1. Enrollment flowchart and patient status 10

3. Table S1. Clinical characteristics of the enrolled study population 11

4. Figure S2. Synthesis and characterization of PGA-C12 nanospheres 12

5. Figure S3. NMR spectra of (A) $hPG(OMs)_{5\%}$ and (B) $hPG(NH_2)_{5\%}$ in D_2O 13

6. Figure S4. NMR spectra of (A) M-PG(OMs)-M in $(CD_3)_2CO$ and (B) M-PGA-M in D_2O 14

7. Figure S5. FTIR spectra of (A) $hPG(N_3)_{5\%}$ and (B) $hPG(NH_2)_{5\%}$ 15

8. Figure S6. FTIR spectra of (A) M-PG(N_3)-M and (B) M-PGA-M 15

9. Figure S7. The size profiles of (A) M-PGA-L, (B) M-PGA-M, (C) M-PGA-S, and (D) PGA-C12 based on the TEM images...16

10. Figure S8. Zeta potential and DLS data of M-PGA-L, M-PGA-M, M-PGA-S and PGA-C12 16

11. Figure S9. UV–visible absorption of PGA and MoS_2 17

12. Figure S10. Biodegradation of MoS_2-L, M-PGA-L, MoS_2-M, M-PGA-M, MoS_2-S and M-PGA-S...18

13. Figure S11. DLS results of M-PGA-M 18

14. Figure S12. Viability of HK-2 cells treated with various concentrations of PGA, M-PGA-L, M-PGA-M, M-PGA-S, PGA-C12, and PAMAM G3..19

15. Figure S13. Biocompatibility and cytocompatibility of M-PGA-M or PGA *in vitro*........19

16. Figure S14. Effect of M-PGA-M on chromatin decondensation in NETs 20

17. Figure S15. Inhibition of oxidative stress in the mixture of platelets and neutrophils by M-PGA-M 21

18. Figure S16. The biodistribution of M-PGA-M in the kidneys of LPS-induced AKI in mice...22

19. Figure S17. The protective effect of M-PGA-M in LPS-induced AKI in mice 23

20. Figure S18. The protective effects of M-PGA-M in LPS-CpG induced AKI................... 24

21. Figure S19. Blockage of renal oxidative stress by M-PGA-M 26

22. Figure S20. Mitigation of LPS-induced multiple organ injury by M-PGA-M................. 27

23. Figure S21. Amelioration of ischemia-reperfusion-induced AKI by M-PGA-M in mice..28

24. References 29

1. Experimental details

Equipment. NMR spectra were recorded with a Jeol Eclipse (Jeol, Japan) nuclear magnetic resonance spectrometer (500 MHz). UV–visible absorption spectra were measured using a U-3310 spectrophotometer (Hitachi, Japan). Zeta potential and dynamic light scattering were conducted in phosphate-buffered saline (PBS) with a pH of 7.4 (Malvern NANO ZSPO, UK). Transmission electron microscopy (TEM) imaging was performed with a FEI Tecnai G2 F30 TEM. cfDNA assays were measured with a multi-well plate reader (Bio-Tek, Winooski, USA).

Patient samples: AKI was identified and classified according to the kidney disease improving global outcomes (KDIGO) criteria.[1] The characteristics of the AKI patients (n = 46) from our hospital are described in Supplemental Table S2. Eleven of these patients had biopsy-proven AKI. Healthy volunteers (n = 47) with matching sex and age were selected as controls, and kidney tissues (n = 6) were taken from donor kidneys that were deemed unsuitable for transplantation. Blood samples were collected within 72 hours after AKI diagnosis, and those enrolled AKI patients received conservative management without dialysis, including underling cause of kidney disease, life-threatening complications, nephrotoxic medications, etc. The study was approved by the First Affiliated Hospital of Sun Yat-Sen University Institutional Review Board (Guangzhou, China). All patients and healthy volunteers provided their written informed consent.

Human hemocyte isolation: Venous blood samples from healthy volunteers was drawn into ethylenediaminetetraacetic acid tubes. Human peripheral platelets were isolated and purified using a platelet isolation kit (Solarbio, China) following the manufacturer's protocol. Briefly, blood samples were diluted with an equal volume of PBS and added to the surface of the separation solution. After centrifugation at 1000*g* for 20 minutes, the platelet-rich plasma layer was retained and washed with PBS until the purified platelets were obtained. Human peripheral blood neutrophils were isolated and purified using a MACSxpress® neutrophil isolation kit (Miltenyi Biotec, Germany) according to the manufacturer's protocol.

Hemolysis activity and biocompatibility assessment: The *in vitro* hemolysis assay was conducted on human erythrocytes. The hemolytic activity of M-PGA-M and PGA was examined at various concentrations in the range of 1–100 μg/mL, with water and PBS as the positive and negative controls, respectively. Cells were incubated on a shaker at 37 °C for 3 hours, followed by centrifugation at 4 °C (3000 rpm, 5 minutes). Supernatants were read at 540 nm in a Multiskan FC microplate reader. Results were expressed as percentages of the control. The morphology of the erythrocytes was observed with a microscope (Olympus, Japan). For the cytocompatibility assay, either 100 μg/mL M-PGA-M or PGA was added to a mixture of human platelets and neutrophils (at a ratio of 50:1) and incubated at 37 °C with 5% CO_2 for 4 hours.[2] Cells were fixed, stained with a Wright–Giemsa stain (LEAGENG, China),[3] and observed under a microscope.

Cell culture and treatment: HK-2 cells (American Type Culture Collection) were cultured in DMEM medium with 10% FBS until the cells were 80% confluent. Cells were treated with either LPS (20 μg/ml), LPS with M-PGA-M, or LPS with PGA (2–4 μg/mL) at 37 °C with 5% CO_2 for 12 hours. Then, the medium was replaced with serum-free DMEM to culture for another 12 hours. The supernatants from various groups were used to measure the cfDNA or collected as a conditioned medium. The conditioned medium was added to the mixtures of platelets and neutrophils from healthy volunteers and incubated at 37 °C with 5% CO_2 for 4 hours. Then, the mixed cells were used for immunofluorescent staining of platelets and NETs.

cfDNA binding test in patient samples and cell supernatants: Human blood samples were centrifuged for 10 minutes at 3500 rpm and 4 °C. The supernatants were pipetted into 96-well plates (100 μL per well) and cultured for 30 minutes in the presence or absence of DMEM, M-PGA-M or PGA (2–4 μg/mL). HK-2 cells were plated in transparent 6-well plates at a density of 1×10^6 cells/well and cultured for 24 hours in the presence of the indicated reagents. The supernatants were collected and the cfDNA concentration was determined using a Quant-iT™ PicoGreen™ dsDNA assay kit (Fisher Scientific, USA) according to manufacturer's protocol.

Cytotoxicity assay: HK-2 cells (1×10^4 cells/well) were seeded in a 96-well plate and incubated with increasing concentrations (from 3 to 1000 μg/mL) of either PGA, M-PGA-S, M-PGA-M, M-PGA-L or PAMAM-G3 for 48 and 72 hours. Cell viability was measured using a Cell Counting Kit-8 (CCK-8, Fisher Scientific, USA) according to the manufacturer's protocol.

Phalloidin dying of HK-2 cell F-actin: HK-2 cells (1×10^5/mL) were treated with either M-PGA-M (100 μg/mL) or PGA (100 μg/mL) at 37 °C with 5% CO_2 for 24 hours. Cells were then fixed, permeabilized and blocked, stained with phalloidin-Alexa Fluor 488 (Invitrogen, A12379, USA) and DAPI (Fisher Scientific, USA) overnight. Images were acquired with a ZEISS LSM880 Airyscan.

Quantification of NET formation: NET formation was quantified by detecting DNA release spectrophotometrically with the DNA-binding dye SYTOX Green, as previously described.[4,5] The mixed cells were stained with 500 nM SYTOX Green and the fluorescence intensity was measured at 1, 2 and 4 hours in a microplate reader at an excitation wavelength of 488 nm. The average fluorescence intensity indicated the amount of NET formation.

Animal models: Male C57BL/6 mice aged 8 to 10 weeks weighing 20 to 22 g were purchased from Jiangsu Gem Pharmatech Biotechnology Company (Jiangsu, China). All animal experiments were performed with the approval of the Institutional Animal Care and Use Committee of the Sun Yat-Sen University. A murine model of LPS-induced AKI was performed as previously reported.[6] Animals were randomly divided into the indicated groups and each group consisted of six mice. Mice were intraperitoneally injected with a single dose of LPS at 10 mg/kg and designated as LPS (positive control), LPS + M-PGA-M (10 mg/kg, i.v.) and LPS + PGA (10 mg/kg, i.v.), whereas the others were designated as saline (negative control), M-PGA-M and PGA.

The combination of LPS and CpG (LPS-CpG) induced AKI model in mice was performed and modified from previously described.[7] Briefly, mice were intraperitoneally injected with

LPS (10 mg/kg), and then 1 mg/kg CpG ODN 1826 (the sequence TCCATGACGTTCCTGATGCT, Synbio Technologies, Shanghai, China) were applied via tail vein. Half hour after LPS and CpG challenge, M-PGA-M (10 mg/kg) was administrated by single intravenous injection. Mice were killed at 24 after modeling (n=5/each group). To investigate the effect of M-PGA-M against LPS-CpG-induced mortality, the experiment was repeated in the presence of increasing concentrations of CpG (2 mg/kg, i.v.), and the survival rate in each group (n=7) was assessed throughout the experiment.

For ischemia reperfusion injury, renal pedicles were clamped for 30 min, as previously reported.[8] Sham-operated mice were performed abdominal incision, but without renal pedicle clamping. For treatment, a single-dose of M-PGA-M (10 mg/kg) or PGA (10 mg/kg) was given though intravenous injection 1 hour before modeling. Their littermates injected with saline were sat as controls. Animals were sacrificed at 24 hours.

Biochemistry index and renal histology: Blood samples were collected to determine serum creatinine, blood urea nitrogen, aspartate transaminase, alanine transaminase, creatine kinase and creatine kinase-MB using a Toshiba Automatic Biochemistry Analyzer (Toshiba, Tokyo, Japan). Serum cfDNA was detected using a Quant-iT™ PicoGreen™ dsDNA Assay Kit. Kidney histology was examined on paraffin-embedded sections stained with hematoxylin and eosin (H&E) and periodic acid–Schiff (PAS). To evaluate the tubular injury score, at least 10 random tissue sections per animal were assessed from the PAS staining in a blinded manner by two renal pathologists. Tubular injury was defined as tubular dilatation, tubular atrophy, loss of the brush border, formation of tubular casts and interstitial edema,[9] and semi-quantified according to the area of the tubular lesion as follows: score 0, no tubular damage; score 1, up to 25%; score 2, 25%–50%; score 3, 50%–75%; score 4, more than 75%. Data are the results from at least six mice per group.

Biodistribution of M-PGA-M and PGA: To assess the biodistribution of M-PGA-M and PGA *in vivo*, the M-PGA-M and PGA were labeled with 100 µg Cy5 (Invitrogen) according to the

manufacturer's protocol. The Cy5-labeled M-PGA-M or PGA was intravenously injected into the tail vein of sham mice or mice treated with LPS (1 hour before injection). After injection, groups of six mice were euthanized at various time points (1, 12, 24, 36 and 48 hours). Tissues, including heart, liver, spleen, lung, and kidney, were excised and *ex vivo* imaged using an IVIS Spectrum imaging system (PerkinElmer, USA). Bright-field and fluorescent images of frozen kidney sections at 24 hours after LPS challenge were acquired with an Olympus FV3000 laser scanning microscope.

Immunohistochemistry and immunofluorescence staining: Immunohistochemistry was performed to detect kidney neutrophils using rat anti-mouse Ly6G monoclonal antibodies (dilution 1:500, B6-8C5, Santa Cruz, USA). For each group, six histologic sections were examined and the number of infiltrating neutrophils was quantified by counting five high-powered fields. For immunofluorescence staining, formaldehyde-fixed cells, or frozen kidney sections from humans and animals were permeabilized, blocked and incubated with primary antibodies against CD42b (mouse monoclonal [AK2] to CD42b, dilution 1:200, ab252264, Abcam, UK), CitH3 (rabbit polyclonal to histone H3, dilution 1:200, ab5103, Abcam, UK), followed by incubation with secondary antibodies.[10] Nuclei were counterstained with DAPI. Images were captured using a Zeiss LSM 880 microscope with Airyscan confocal super-resolution. The percentage of positive staining was analyzed and quantified with ImageJ software.

Oxidative stress assay: The mixtures of platelets (200×10^6/mL) and neutrophils (4×10^6/mL) cultured in confocal dishes were treated with corresponding the conditioned medium at 37 °C with 5% CO_2 for 4 hours. Cells or frozen mouse kidney sections were fixed, permeabilized and blocked. The generation of ROS was detected using a DCFH-DA assay (Dojindo, Japan)[2] according to the manufacturer's protocol. Super-resolution images were obtained using a Zeiss LSM 880 microscope. ROS-positive cells were counted in 10 non-overlapping high-power fields (original magnification × 40). The levels of malonaldehyde, superoxide dismutase and

glutathione in cells or renal tissues were examined using commercial kits (Solarbio, China) following the manufacturer's protocol.

Statistical analysis: Data were expressed as the means ± SD. The differences between groups were assessed by either the Student's t-test (for simple two-sample comparison) or one-way analysis of variance (ANOVA) with Tukey's post hoc test (for multiple comparison). Linear correlation and regression analysis were used to determine the correlation between cfDNA and other indicators. A value of $P < 0.05$ was considered statistically significant. The Kaplan-Meier curves were used to compare the survival differences.

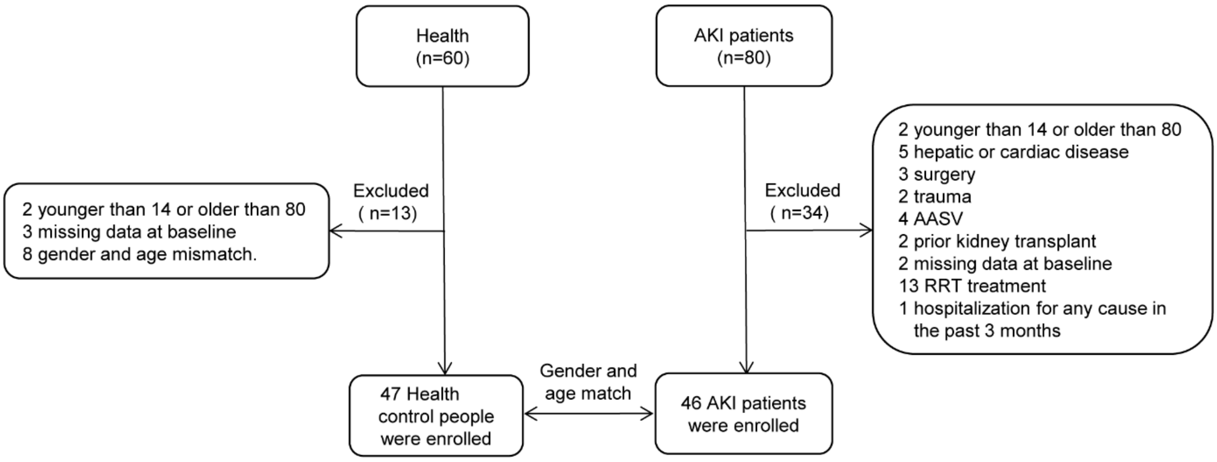

Figure S1. Enrollment flowchart and patient status

Table S1. Clinical characteristics of the enrolled study population

Characteristic	Healthy (n = 47)	AKI (n = 46)	P-value
Age (y)	47.06 ± 16.68	52.34 ± 17.33	0.146
Sex (Male/Female)	33/14	31/15	0.769
Sepsis (%)	—	30.43	—
Nephrotic Syndrome (%)	—	23.91	—
Lupus Nephritis (%)	—	21.74	—
Chemotherapeutic drug associated AKI (%)	—	15.22	—
Malignant Hypertension (%)	—	4.36	—
Obstructive Nephropathy (%)	—	2.17	—
Tsutsugamushi Diseases (%)	—	2.17	—
Serum creatinine (μmoI/L)	69.77 ± 11.81	341.09 ± 195.48	0.000
Neutrophils (×10^9/L)	—	7.27 ± 3.99	—
cfDNA (ng/mL)	177.40 ± 50.21	362.51 ± 145.89	0.000
The amount of cfDNA (ng/mL) for the different clinicla AKI entities			
Sepsis	—	401.68 ± 184.86	—
Nephrotic Syndrome	—	326.95 ± 106.50	—
Lupus Nephritis	—	384.51 ± 148.10	—
Chemotherapeutic Drug	—	299.59 ± 109.41	—
Others	—	389.30 ± 200.83	—

Values are expressed as the mean ± SD or number (percent). $P < 0.05$ was considered as statistically significant.

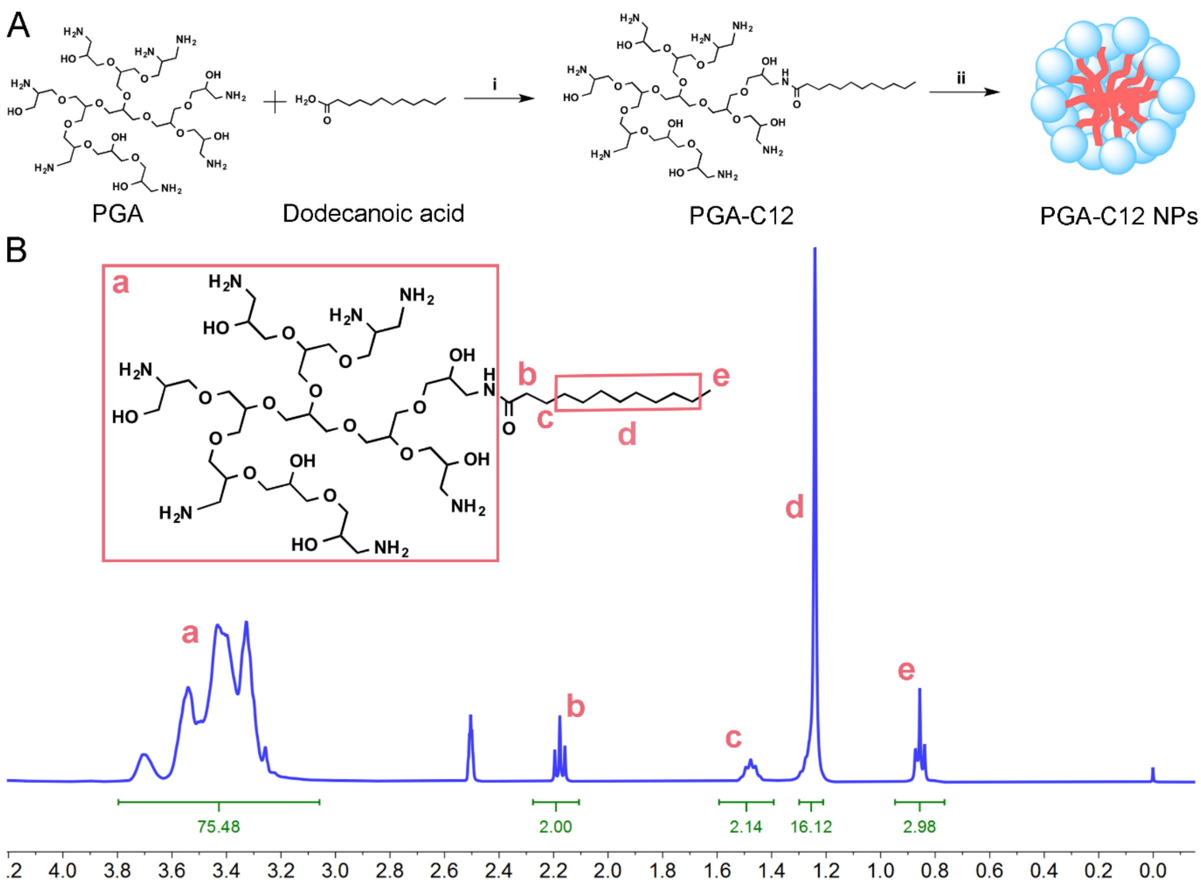

Figure S2. Synthesis and characterization of PGA-C12 nanospheres. A) Synthesis of PGA-C12 nanoparticles. Step i) 1, dissolution of EDC.HCl and NHS in DMF solution overnight at room temperature; 2, dialysis in Milli-Q (MWCO = 2 KDa), overnight; 3, lyophilization. Step ii) 1, dissolution in DMSO; 2, dropwise addition to Milli-Q. B) ^{1}H NMR spectrum of PGA-C12 in D6-DMSO. The average number of protons in one PGA molecule ($M_n \approx 5000$ g/mol) was around 338 (position a), and the number of protons in the b position was 2 in one dodecanoic acid (DA) molecule. The molecular ratio of DA and PGA in PGA-C12 was calculated as follows: $\mathrm{DA/PGA} = \frac{2/2}{75.5/338} = 4.48$.

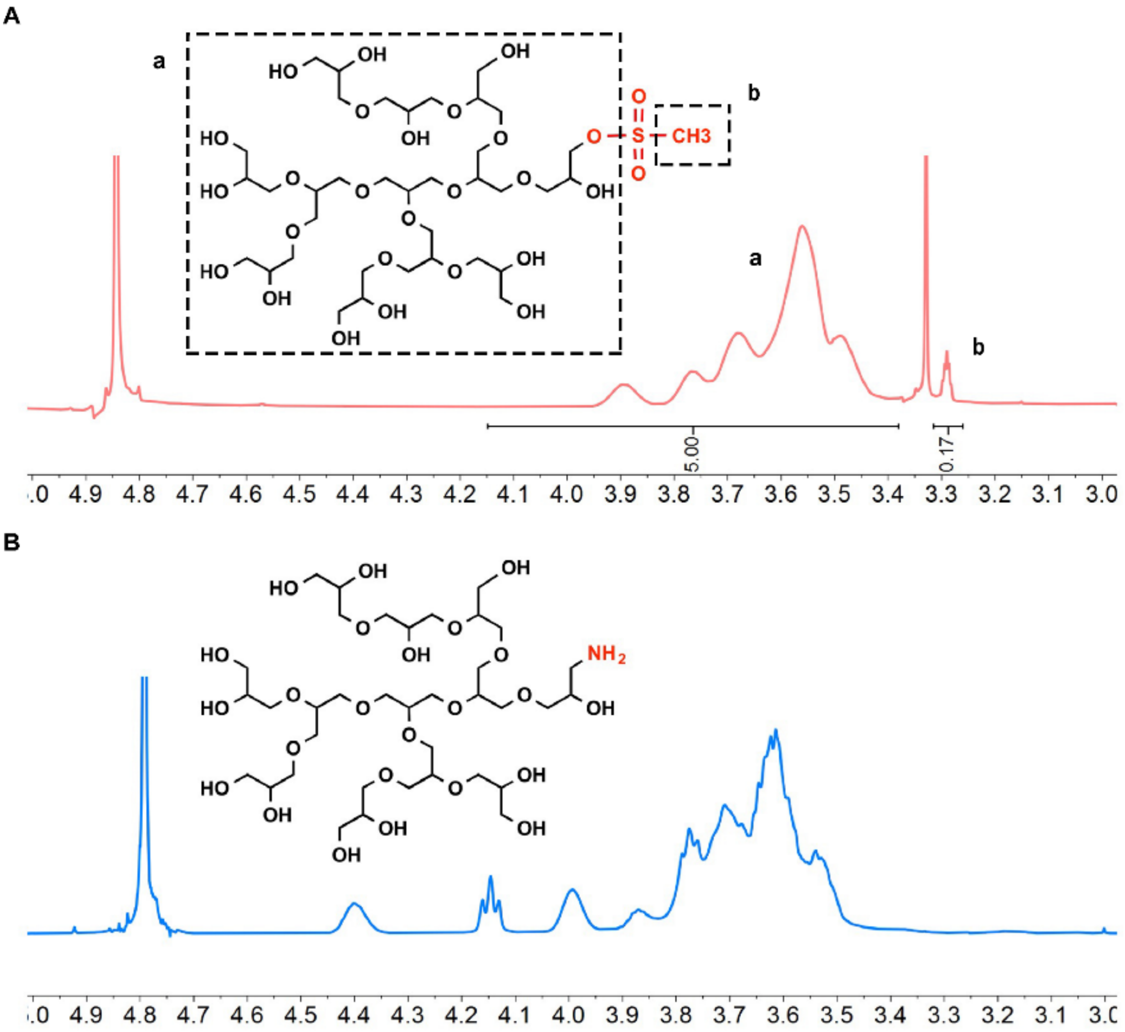

Figure S3. NMR spectra of (A) hPG(OMs)$_{5\%}$ and (B) hPG(NH$_2$)$_{5\%}$ in D_2O. This confirmed that 5% of hydroxyl groups were replaced by methanesulfonyl groups on hPG(OMs)$_{5\%}$, which were, in turn, reduced to amino groups to give hPG(NH$_2$)$_{5\%}$. The signals at 3.4–4.4 ppm and 3.1–3.25 ppm were attributed to the protons of the hPG backbone and the methyl group of methanesulfonyl, respectively.

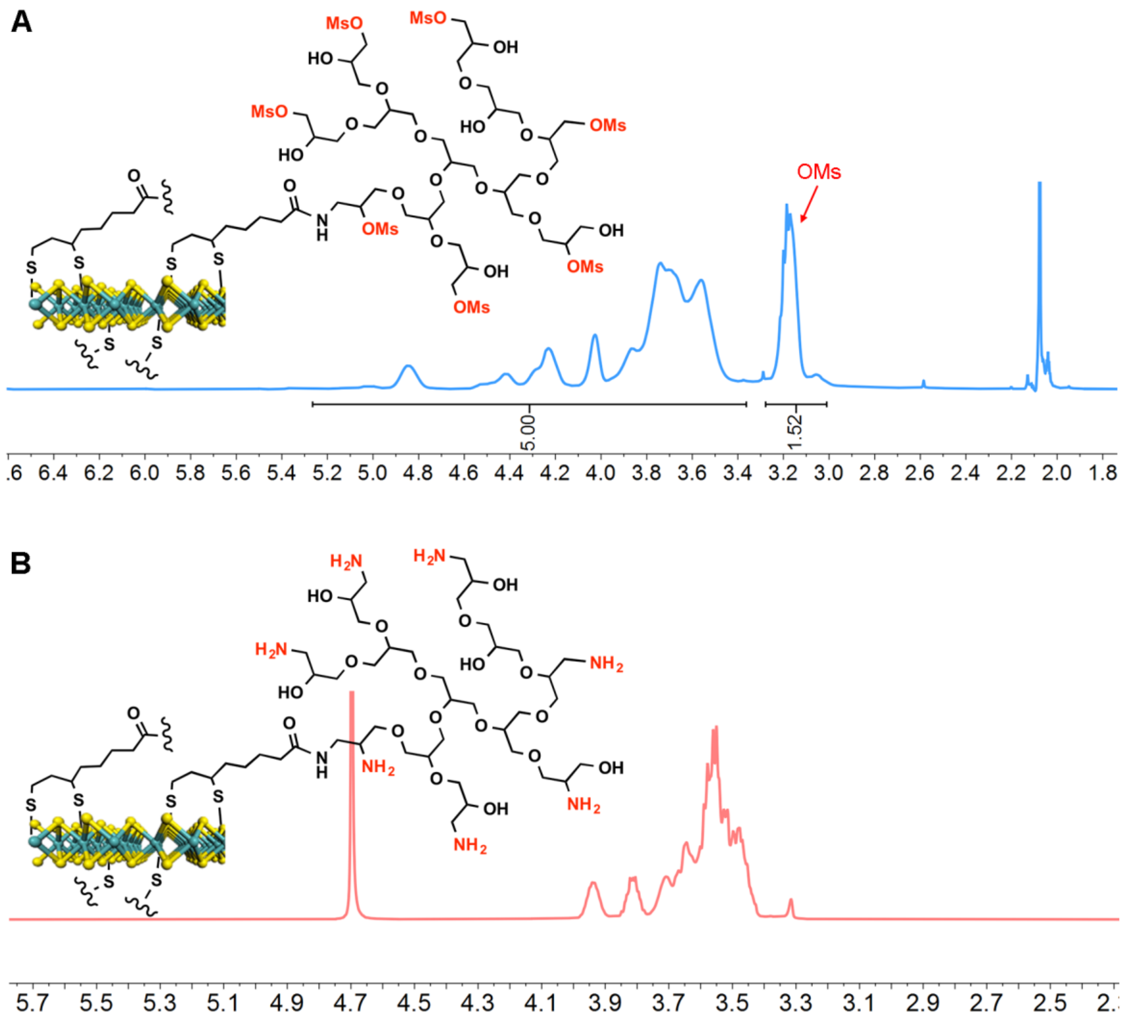

Figure S4. NMR spectra of (A) M-PG(OMs)-M in $(CD_3)_2CO$ and (B) M-PGA-M in D_2O. This confirmed that approximately 50% of the hydroxyl groups were replaced by methanesulfonyl groups on M-PG(OMs)-M, and that all methanesulfonyl groups were then reduced to amino groups to give M-PGA-M. The signals at 3.4–4.4 ppm and 3.1–3.25 ppm were attributed to the protons of the hPG backbone and methyl group of methanesulfonyl, respectively.

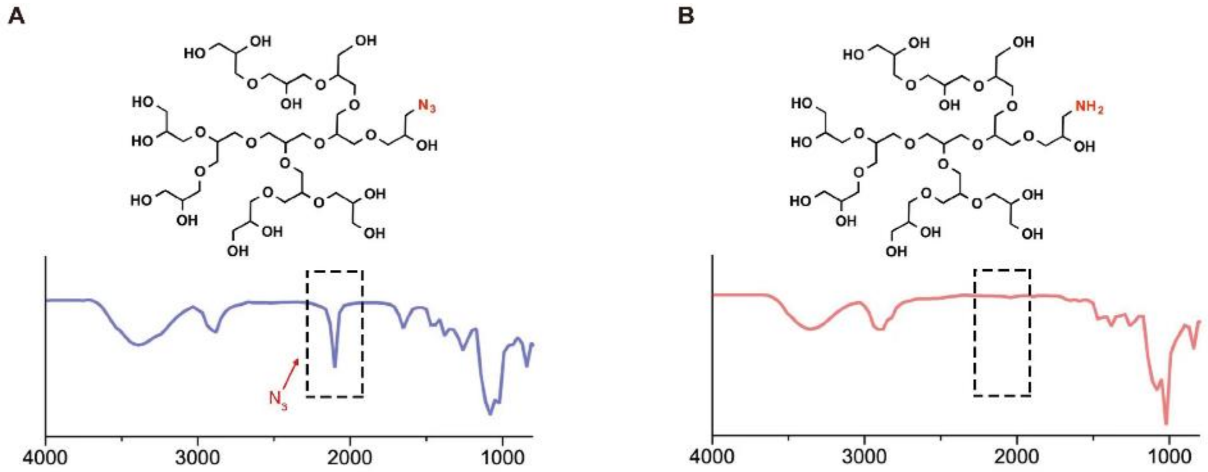

Figure S5. FTIR spectra of (A) hPG(N_3)$_{5\%}$ and (B) hPG(NH_2)$_{5\%}$. The appearance of a new absorbance band at 2100 cm^{-1} for hPG(OMs)$_{5\%}$ after reaction with sodium azide confirmed the successful azidation of hPG. The reduction of hPG(N_3)$_{5\%}$ to hPG(NH_2)$_{5\%}$ was substantiated by the disappearance of the azide peak.

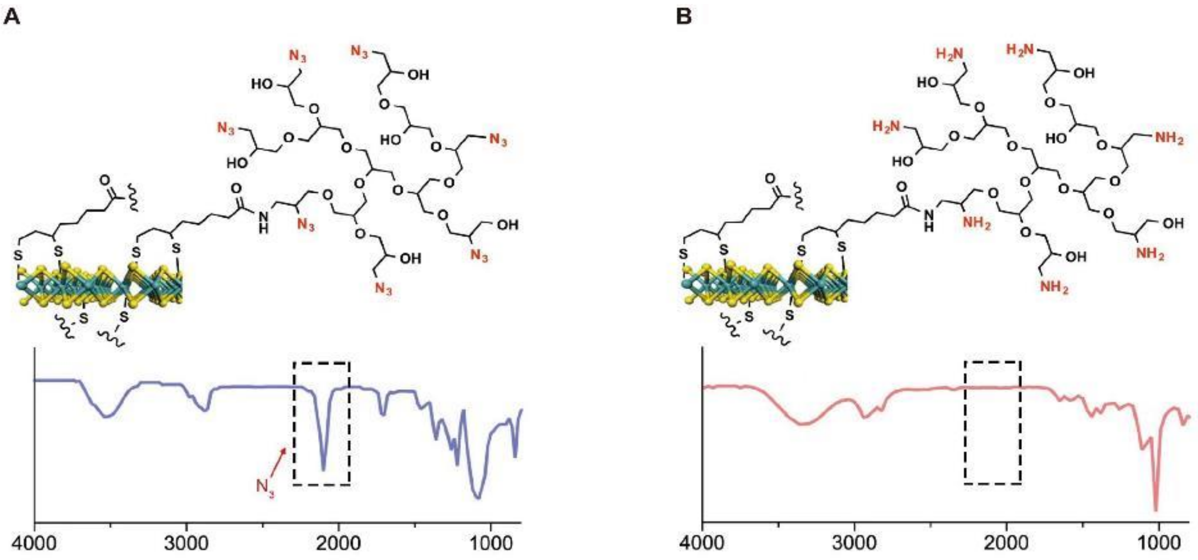

Figure S6. FTIR spectra of (A) M-PG(N_3)-M and (B) M-PGA-M. The new absorbance band at 2100 cm^{-1} for M-PG(N_3)-M after reaction with sodium azide confirmed the successful azidation of M-PG-M. The disappearance of the azide peak for M-PGA-M indicated the reduction of azide groups to amino groups.

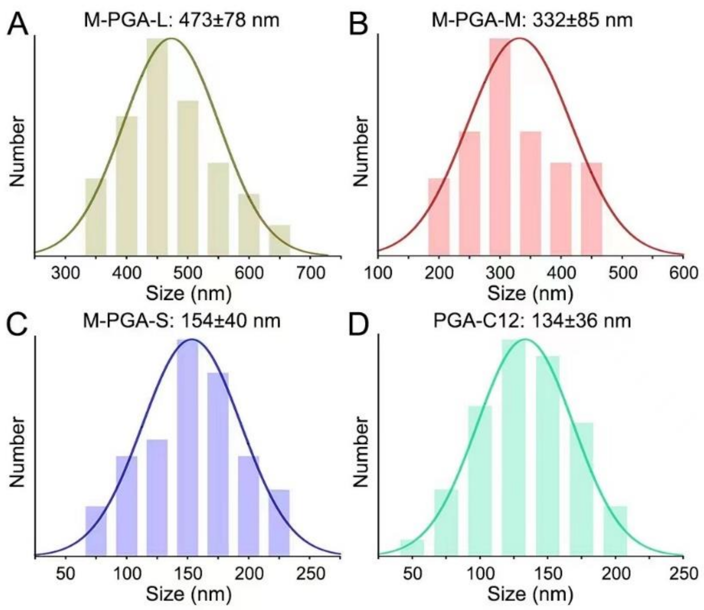

Figure S7. The size profiles of (A) M-PGA-L, (B) M-PGA-M, (C) M-PGA-S, and (D) PGA-C12 based on the TEM images.

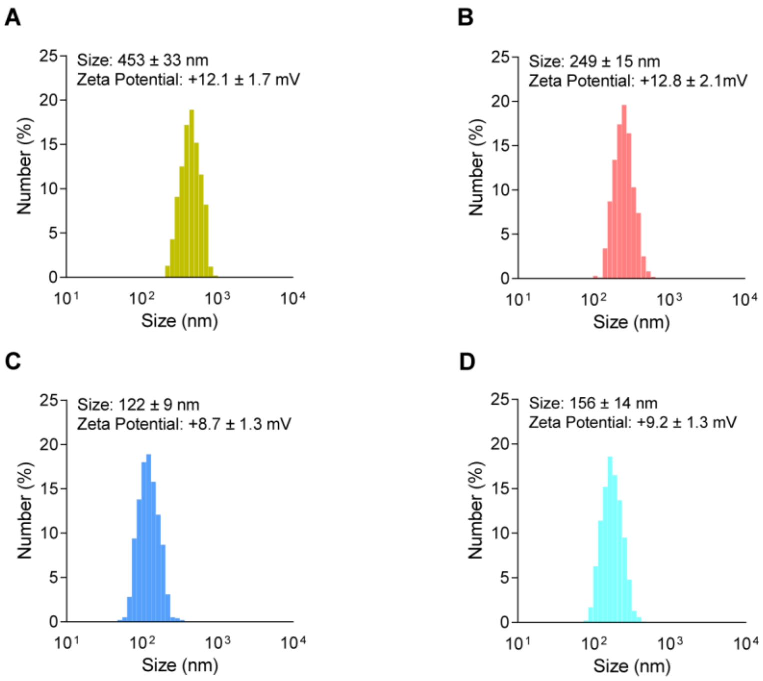

Figure S8. Zeta potential and DLS data of (A) M-PGA-L, (B) M-PGA-M, (C) M-PGA-S and (D) PGA-C12 in PBS (pH 7.4).

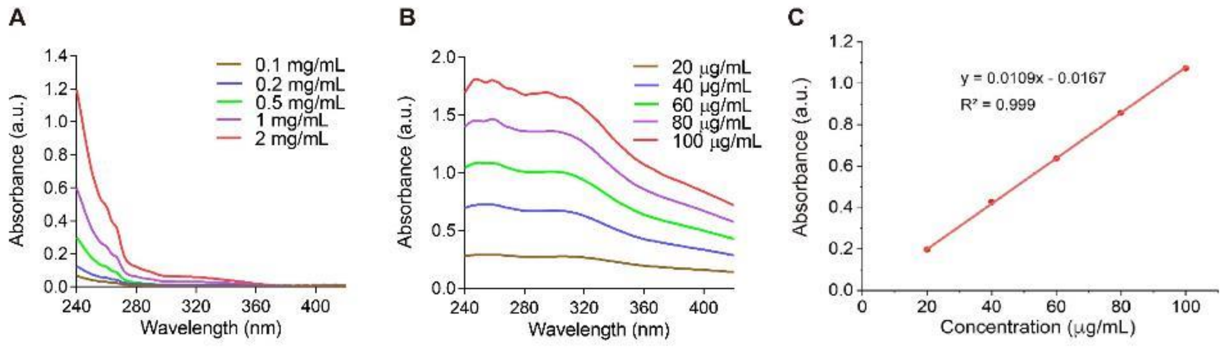

Figure S9. UV–visible absorption of PGA and MoS_2. A) UV–visible absorbance of PGA at different concentrations from 0.1 to 2 mg/mL. B) UV–visible absorbance of MoS_2 at different concentrations from 20 to 100 μg/mL. C) Standard curve of MoS_2 with absorbance at 360 nm. The absorbance values at 360 nm of M-PGA-L, M-PGA-M and M-PGA-S (200 μg/mL) solutions were 0.432, 0.361 and 0.324, giving MoS_2 concentrations of 41.2, 34.7 and 31.3 μg/mL, respectively. The MoS_2 content of M-PGA-L, M-PGA-M and M-PGA-S was 20.6%, 17.4% and 15.7%, respectively. Therefore, the polymer content of M-PGA-L, M-PGA-M and M-PGA-S was 79.4%, 82.6% and 84.3%, respectively.

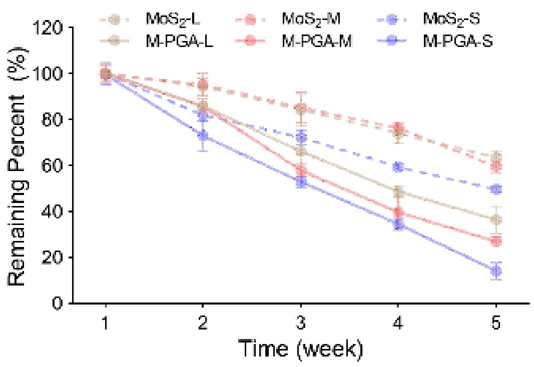

Figure S10. Biodegradation of MoS_2-L, M-PGA-L, MoS_2-M, M-PGA-M, MoS_2-S and M-PGA-S in PBS (7.4) at 37 °C for 4 weeks. The biodegradation was quantified as the remaining percentage of nanomaterials based on the UV adsorption at 360 nm.

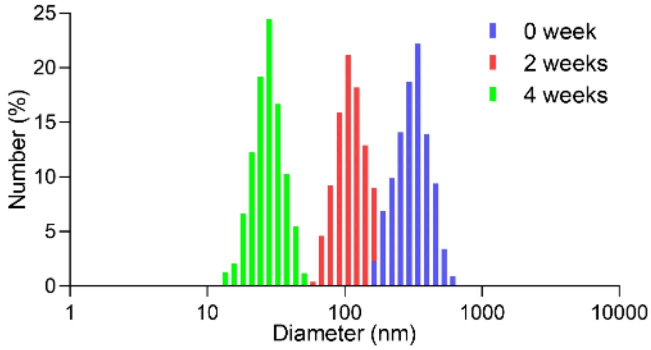

Figure S11. DLS results of M-PGA-M before and after 2 or 4 weeks' incubation in PBS (7.4) at 37 °C.

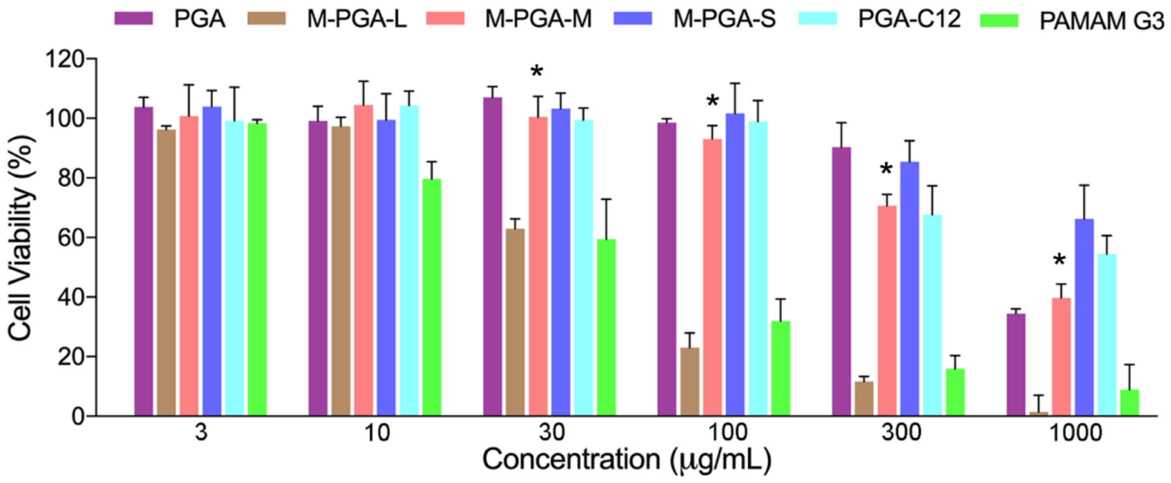

Figure S12. Viability of HK-2 cells treated for 72 hours with various concentrations of PGA, M-PGA-L, M-PGA-M, M-PGA-S, PGA-C12, and PAMAM G3. *P < 0.05, compared with M-PGA-L or PAMAM-G3.

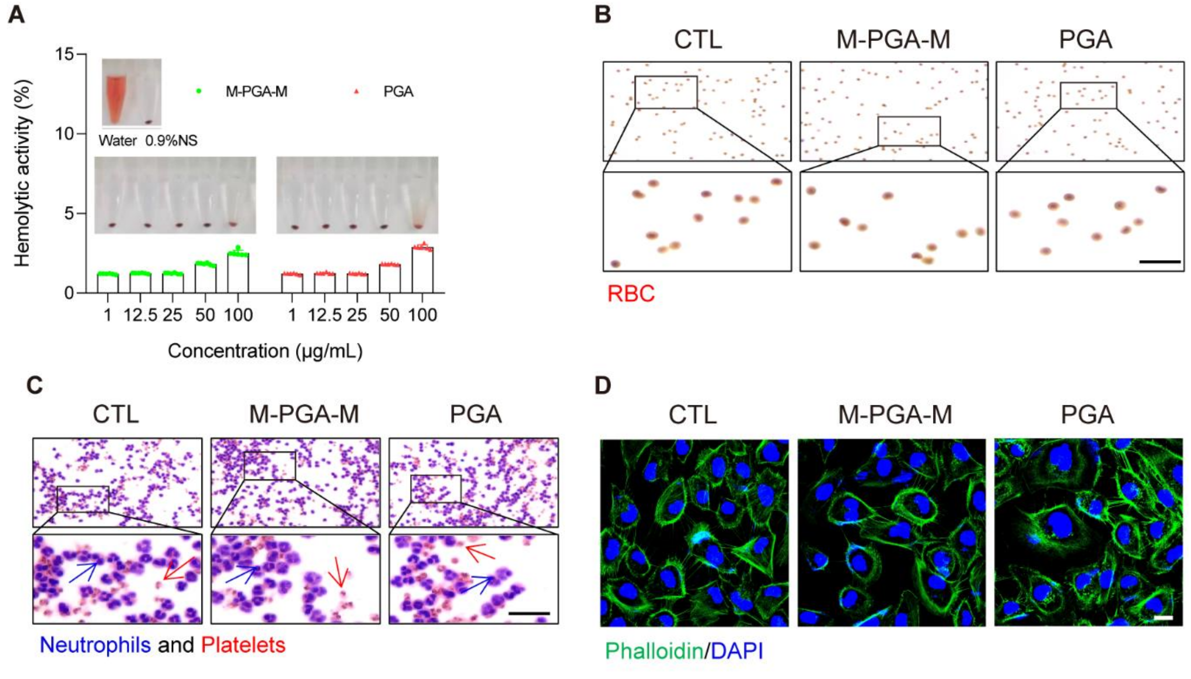

Figure S13. Biocompatibility and cytocompatibility of M-PGA-M or PGA *in vitro*. A) Human red blood cell (RBC) hemolysis test of M-PGA-M and PGA at different concentrations. The insets are digital photographs of the M-PGA-M and PGA solutions. The data are from three independent experiments. (B–D) Representative images of different human cells before and

after incubation with M-PGA-M (100 μg/mL) or PGA (100 μg/mL). B) Microscope images of the RBCs. Scale bars: 50 μm. C) Wright–Giemsa staining of a suspension of neutrophils and platelets. The blue arrows and red arrows indicate neutrophils and platelets, respectively. Scale bars: 50 μm. D) Cytoskeleton staining of HK-2 cells. Scale bars: 20 μm.

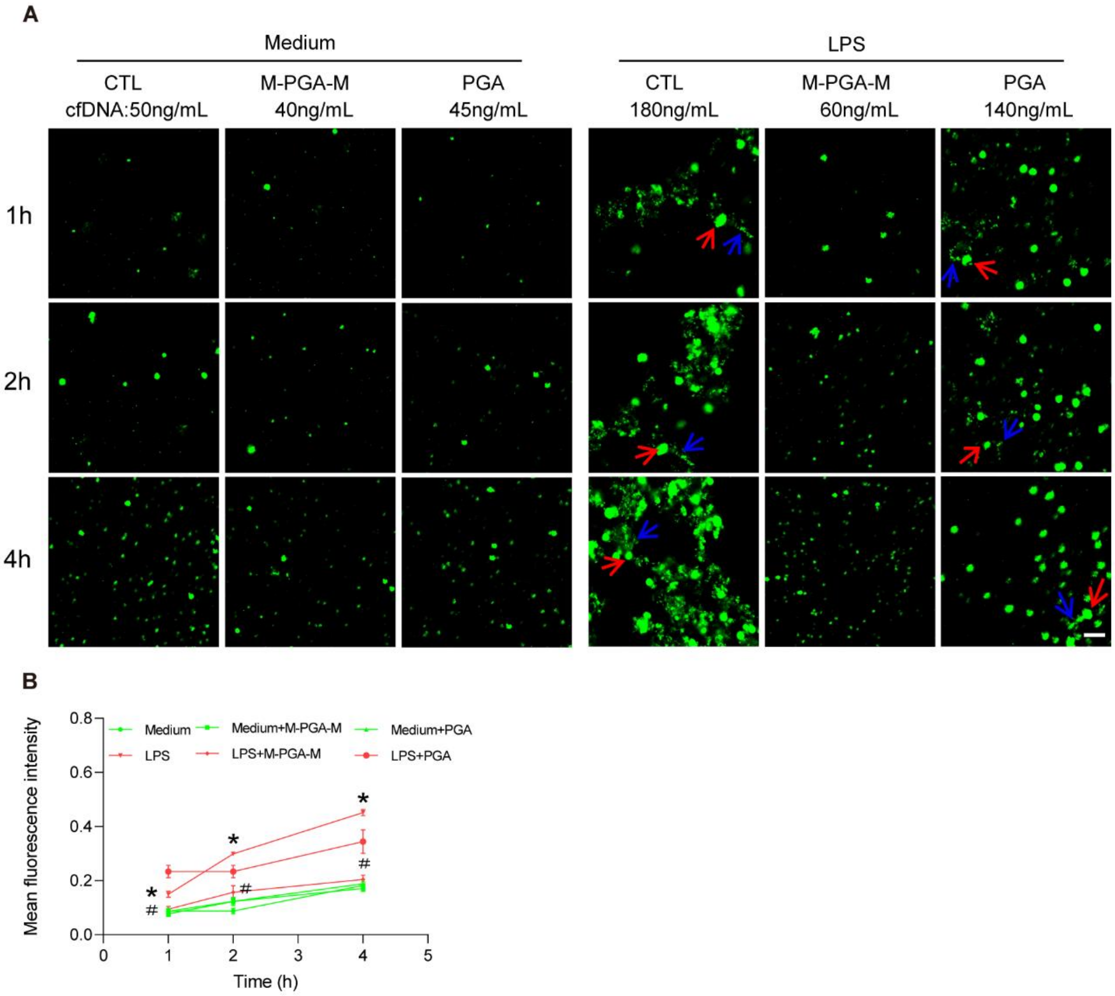

Figure S14. Effect of M-PGA-M on chromatin decondensation in NETs. A) Dynamic analysis of condensed chromatin (red arrows) decondensed chromatin (blue arrows) by Sytox Green staining. Scale bar: 50 μm. B) Sytox Green fluorescence intensity measured with a microplate reader. The data shown represent three independent experiments. The differences were assessed via Kruskal–Wallis and Mann–Whitney U tests (*$P < 0.05$, compared with the medium; $^{\#}P <$ 0.05, compared with LPS alone or LPS + PGA).

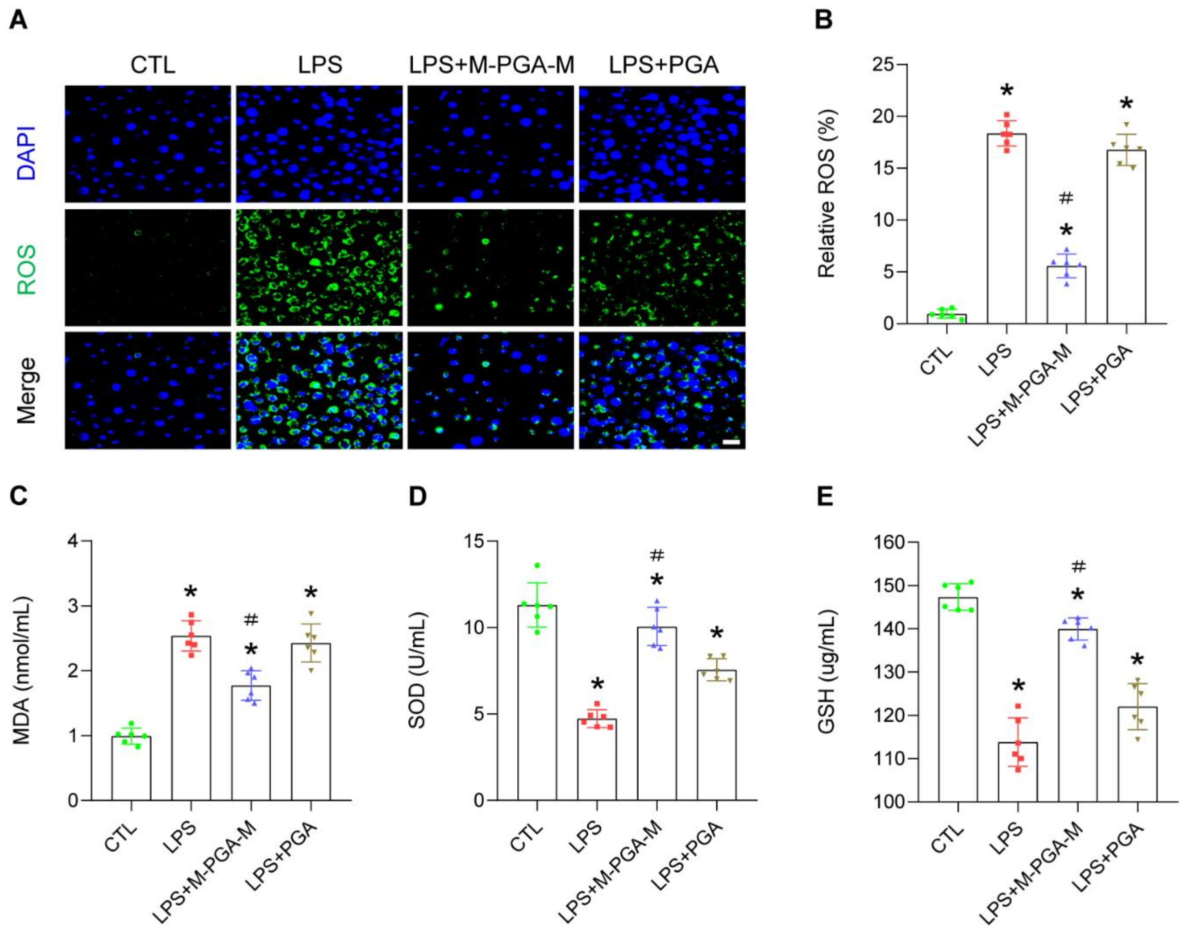

Figure S15. Inhibition of oxidative stress in the mixture of platelets and neutrophils by M-PGA-M. Mixture of platelets and neutrophil stimulated with a conditioned medium. A) Representative ROS fluorescent staining for different treatment conditions. Scale bar: 20 µm. B) Quantitative data for the ROS levels in (A). C–E) Levels of malonaldehyde (MDA) (C), superoxide dismutase (SOD) (D) and glutathione (GSH) (E) under the different treatment conditions. The data are expressed as the means ± SD and the categorical variables are presented as percentages. All experiments were performed in triplicate. The differences were assessed via Kruskal–Wallis and Mann–Whitney U tests in (B) and one-way ANOVA with Tukey's multiple comparison tests in (C–E) (*$P < 0.05$, compared with the control (CTL); $^{\#}P < 0.05$, compared with LPS alone or LPS + PGA).

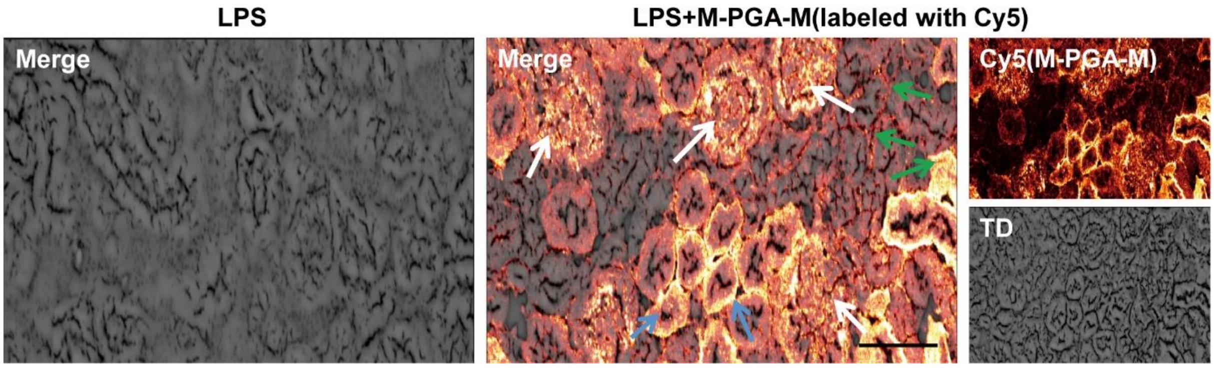

Figure S16. The biodistribution of M-PGA-M in the kidneys of LPS-induced AKI in mice. Representative fluorescent and bright-field images of frozen renal sections. Scale bars: 200 µm. The blue, green, and white arrows indicated renal tubular epithelial cells, interstitial space and glomerulus, respectively.

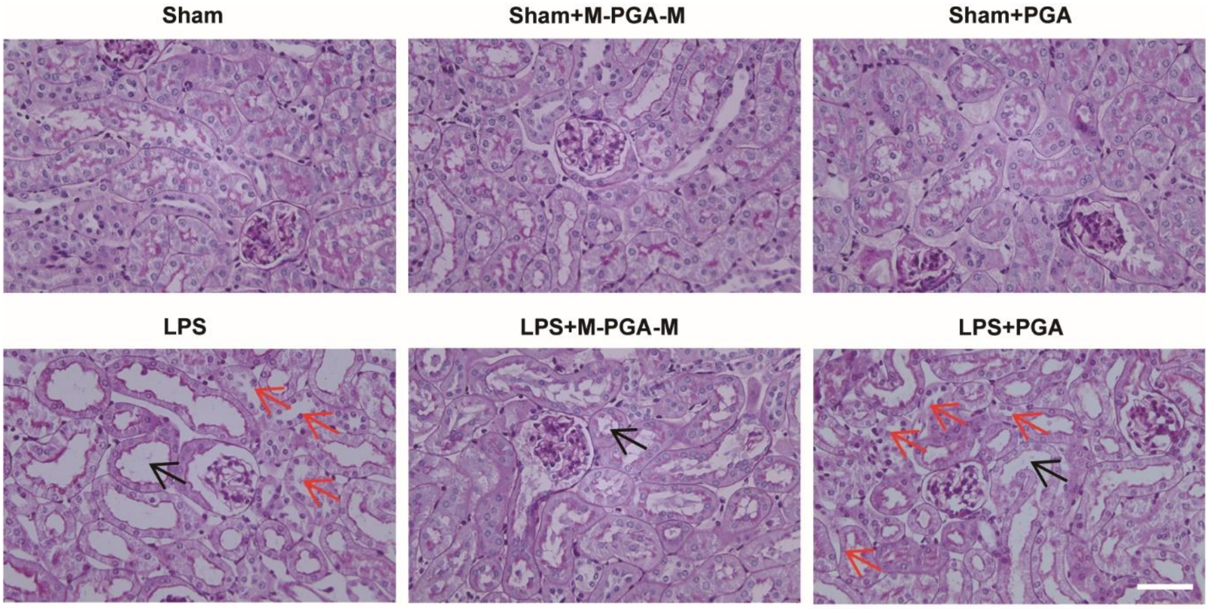

Figure S17. The protective effect of M-PGA-M in LPS-induced AKI in mice. Representative images of renal PAS staining. Scale bars: 100 μm. The black and red arrows indicate dilated tubules and the loss of the proximal tubular brush-border membrane, respectively.

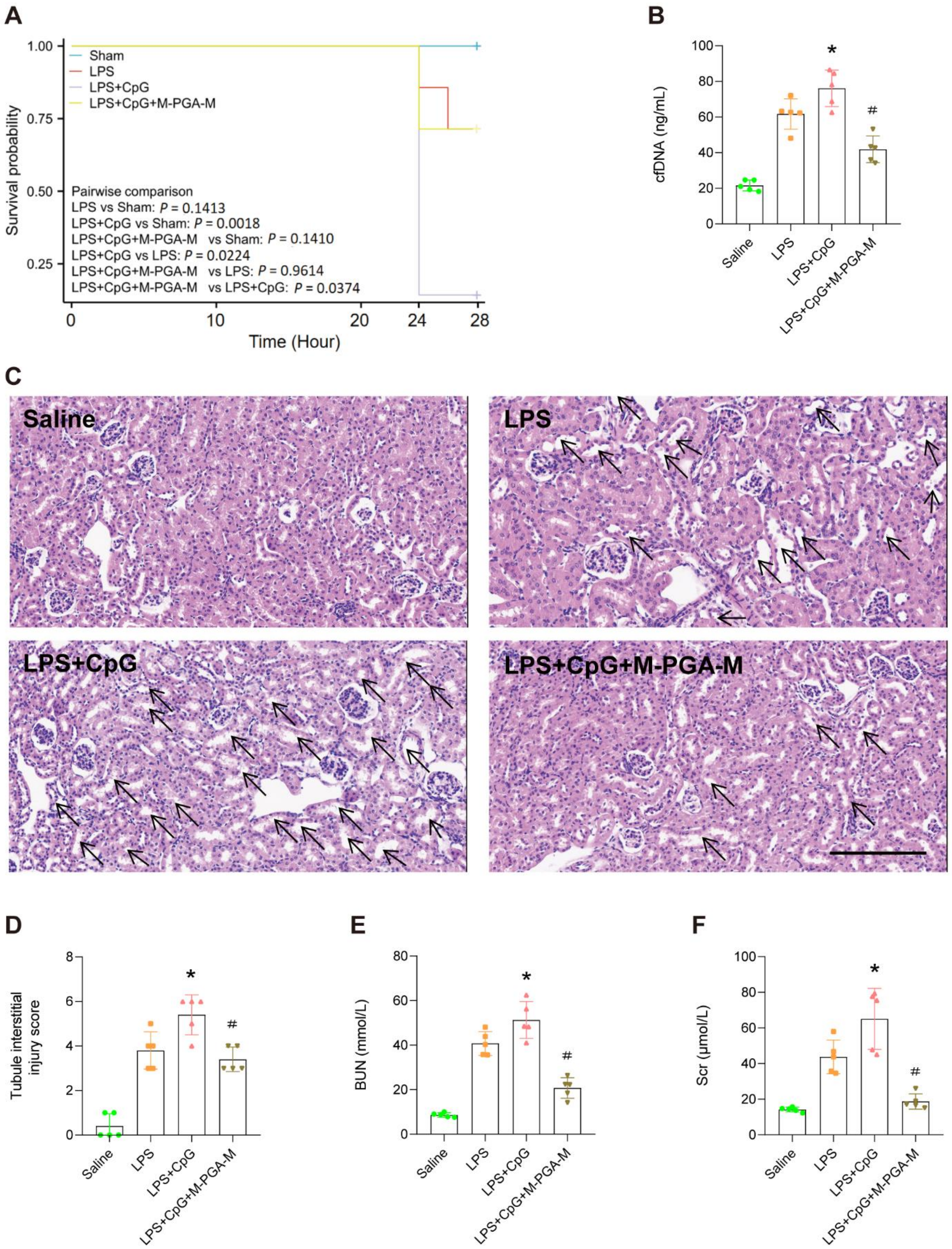

Figure S18. M-PGA-M protects mice against LPS-CpG induced AKI. A) The LPS-induced AKI model was constructed in mice, followed by tail vein injection of 10 mg/kg M-PGA-M after 30 min of CpG challenge. Survival was monitored for 28 hours (n=7 mice per group; Kaplan-Meier survival analysis). B) Changes in serum cfDNA levels in the indicated group at

24 hours after treatment (n=5 mice per group). C) Representative images of renal H&E staining. The black arrows indicate dilated tubules. Scale bars: 200 μm. D) Quantification of tubulointerstitial damage. E–F) Levels of blood urea nitrogen (BUN) (E) and creatinine (SCr) (F) in LPS-CpG induced AKI mice with or without M-PGA-M at 24 hours. The data are expressed as the means ± SD. The differences were assessed via one-way ANOVA with Tukey's multiple comparison test (*P < 0.05, compared with LPS; $^{\#}P$ < 0.05, compared with LPS+CpG).

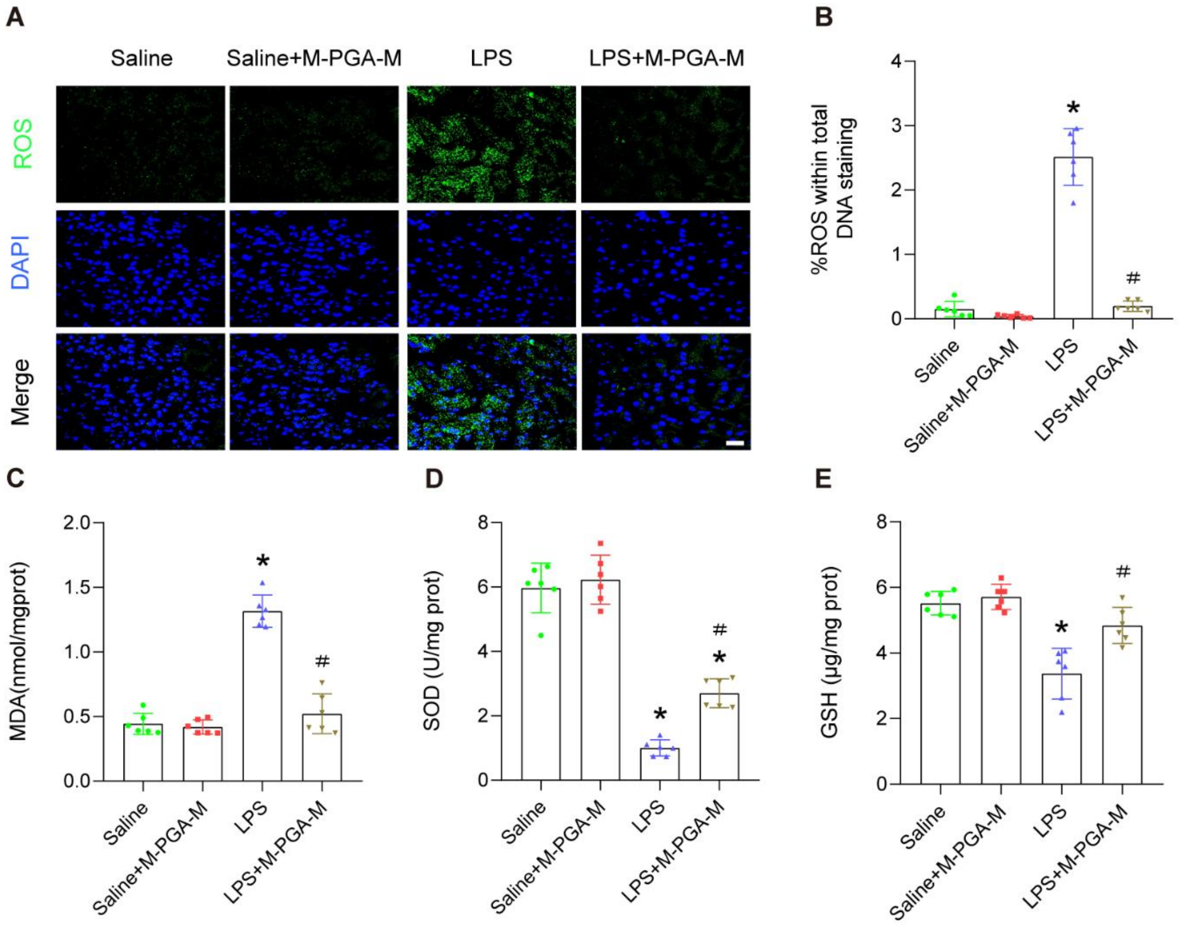

Figure S19. Blockage of renal oxidative stress by M-PGA-M. Kidney tissues were collected 24 hours after different treatments. A) Representative ROS fluorescent staining of kidneys. Scale bar: 20 μm. B) Quantitative data for the ROS levels in (A). C–E) levels of MDA (C), SOD (D) and GSH (E) in the kidneys of indicated groups. The data are expressed as the means ± SD and the categorical variables are presented as percentages. The differences were assessed via Kruskal–Wallis and Mann–Whitney U tests in (B) and one-way ANOVA with Tukey's multiple comparison tests in (C–E) (*P < 0.05, compared with saline; $^{\#}P$ < 0.05, compared with LPS).

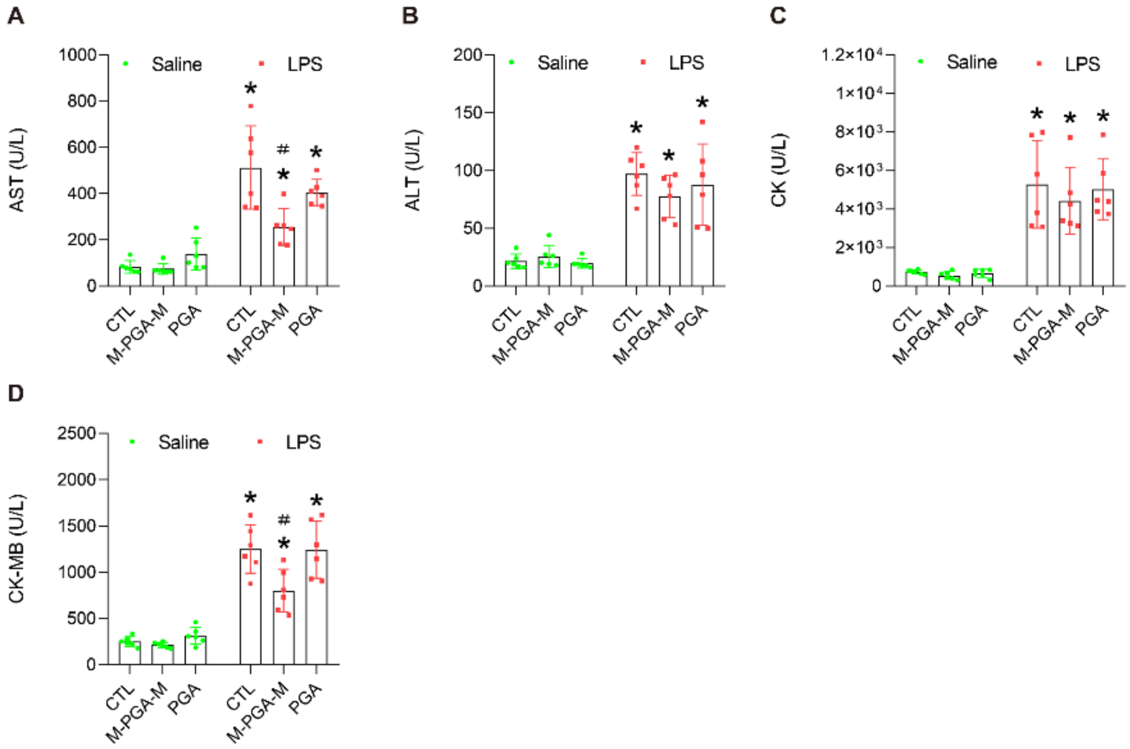

Figure S20. Mitigation of LPS-induced multiple organ injury by M-PGA-M. A–D) Blood samples collected 24 hours after different treatments. Serum levels of aspartate transaminase (AST) (A), alanine transaminase (ALT) (B), creatine kinase (CK) (C) and creatine kinase-MB (CK-MB) (D) in indicated groups. The data are expressed as the means ± SD (n = 6). The differences were assessed via Kruskal–Wallis and Mann–Whitney U test (*$P < 0.05$, compared with saline; $^{\#}P < 0.05$, compared with LPS alone or LPS+PGA).

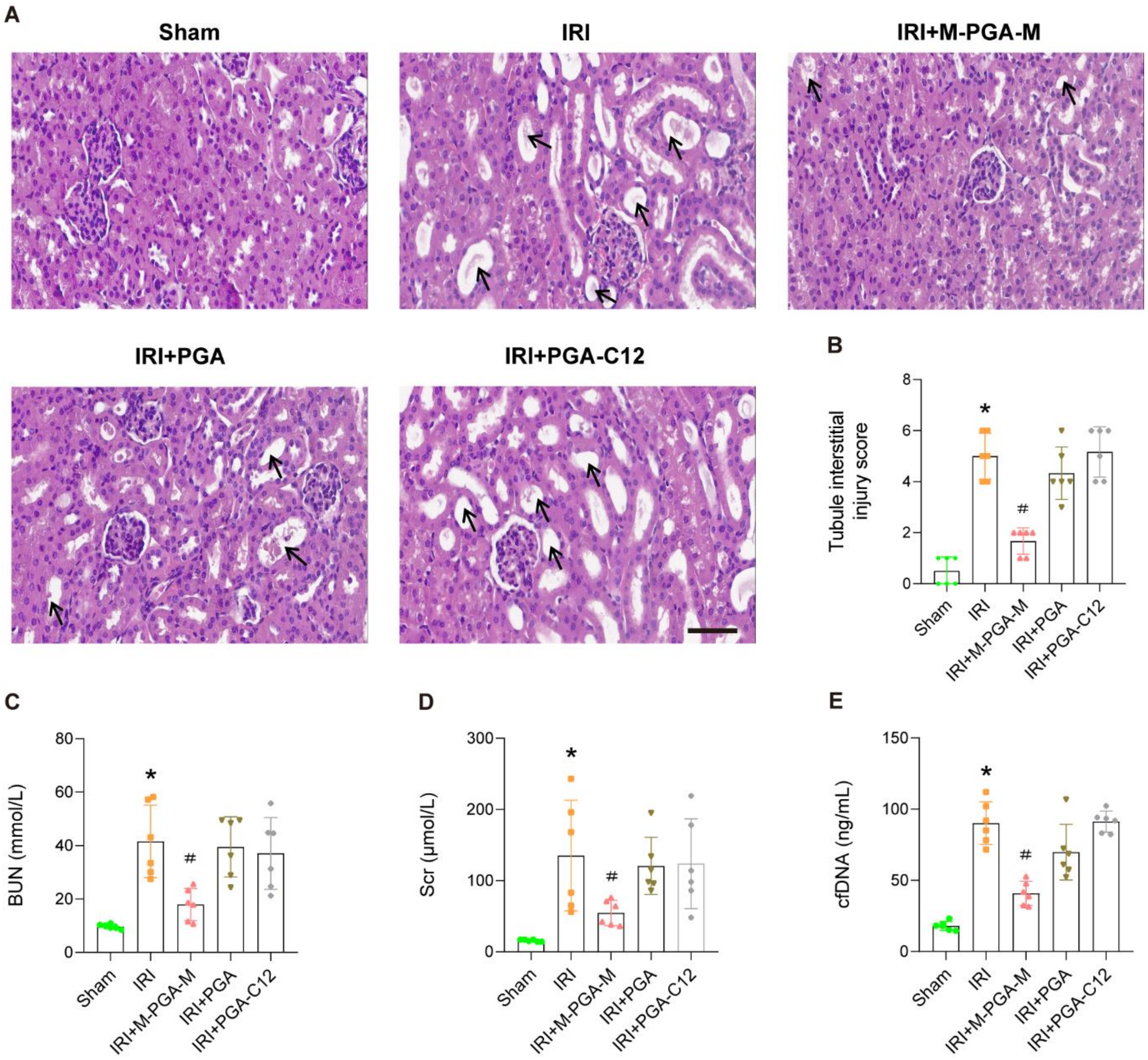

Figure S21. Amelioration of ischemia-reperfusion-induced AKI by M-PGA-M in mice. A) Representative images of renal H&E staining. Scale bars: 50 μm. The black arrows indicate dilated tubules. B) Quantification of tubulointerstitial damage. C–E) Changes in blood urea nitrogen (BUN) (C), serum creatinine (SCr) (D) and cfDNA (E) levels in ischemia-reperfusion-induced AKI mice with or without M-PGA-M, PGA or PGA-C12 at 24 hours. The data are expressed as the means ± SD (n = 6). The differences were assessed via one-way ANOVA with Tukey's multiple comparison test ($^*P < 0.05$, compared with Sham; $^{\#}P < 0.05$, compared with IRI).

References

[1] *Kidney Int Suppl (2011)* **2012**, *2*, 19.
[2] X. Zhao, L. Y. Wang, J. M. Li, L. M. Peng, C. Y. Tang, X. J. Zha, K. Ke, M. B. Yang, B. H. Su, W. Yang, *Advanced science (Weinheim, Baden-Wurttemberg, Germany)* **2021**, *8*, e2101498.
[3] B. Moreira-Pinto, L. Costa, E. Felgueira, B. M. Fonseca, I. Rebelo, *Antioxidants (Basel, Switzerland)* **2021**, *10*,
[4] A. Clementi, G. M. Virzì, A. Brocca, S. Pastori, M. de Cal, S. Marcante, A. Granata, C. Ronco, *Blood Purif.* **2016**, *41*, 34.
[5] Q. Ou, J. Q. Fang, Z. S. Zhang, Z. Chi, J. Fang, D. Y. Xu, K. Z. Lu, M. Q. Qian, D. Y. Zhang, J. P. Guo, W. Gao, N. R. Zhang, J. P. Pan, *Nat Commun* **2021**, *12*, 3481.
[6] Y. Ding, Y. Zheng, J. Huang, W. Peng, X. Chen, X. Kang, Q. Zeng, *Int. Immunopharmacol.* **2019**, *71*, 336.
[7] X. Meng, W. Sun, Y. Ren, Y. Xiao, P. Zhao, W. Lu, L. Hua, L. Wang, L. Wang, Y. Yu, *Mol. Immunol.* **2017**, *90*, 74.
[8] M. T. Tran, Z. K. Zsengeller, A. H. Berg, E. V. Khankin, M. K. Bhasin, W. Kim, C. B. Clish, I. E. Stillman, S. A. Karumanchi, E. P. Rhee, S. M. Parikh, *Nature* **2016**, *531*, 528.
[9] K. Wu, W. Lei, J. Tian, H. Li, *BMC Nephrol* **2014**, *15*, 14.
[10] M. P. Jansen, D. Emal, G. J. Teske, M. C. Dessing, S. Florquin, J. J. Roelofs, *Kidney Int.* **2017**, *91*, 352.
